# The journey of cardosin A in young Arabidopsis seedlings leads to evidence of a Golgi-independent pathway to the protein storage vacuole

**DOI:** 10.3389/fpls.2023.1085898

**Published:** 2023-07-07

**Authors:** Cláudia Pereira, Vanessa Vieira, José Pissarra, Susana Pereira

**Affiliations:** GreenUPorto—Sustainable Agrifood Production Research Centre/Inov4Agro, Department of Biology, Faculty of Sciences, University of Porto, Porto, Portugal

**Keywords:** cardosin A, aspartic proteinase, plant-specific insert, vacuole, unconventional route, vacuolar sorting determinant

## Abstract

The aspartic proteinase cardosin A is a vacuolar enzyme found to accumulate in protein storage and lytic vacuoles in the flowers and protein bodies in the seeds of the native plant cardoon. Cardosin A was first isolated several decades ago and has since been extensively characterized, both in terms of tissue distribution and enzyme biochemistry. In the native system, several roles have been attributed to cardosin A, such as reproduction, reserve mobilization, and membrane remodeling. To participate in such diverse events, cardosin A must accumulate and travel to different compartments within the cell: protein storage vacuoles, lytic vacuoles, and the cytoplasmic membrane (and eventually outside the cell). Several studies have approached the expression of cardosin A in *Arabidopsis thaliana* and *Nicotiana tabacum* with promising results for the use of these systems to study of cardosin A trafficking. A poly-sorting mechanism has been uncovered for this protein, as two different vacuolar sorting determinants, mediating different vacuolar routes, have been described. The first is a conventional C-terminal domain, which delivers the protein to the vacuole via the Golgi, and the second is a more unconventional signal—the plant-specific insert (PSI)—that mediates a Golgi-independent route. The hypothesis that these two signals are activated according to cell needs and in organs with high metabolic activity is investigated here. An Arabidopsis line expressing cardosin A under an inducible promoter was used to understand the dynamics of cardosin A regarding vacuolar accumulation during seed germination events. Using antibodies against different regions of the protein and combining them with immunofluorescence and immunocytochemistry assays in different young seedling tissues, cardosin A was detected along the secretory pathway to the protein storage vacuole, often associated with the endoplasmic reticulum. More interestingly, upon treatment with the drug Brefeldin A, cardosin A was still detected in protein storage vacuoles, indicating that the intact protein can bypass the Golgi in this system, contrary to what was observed in *N. tabacum*. This study is a good starting point for further research involving the use of fluorescent fusions and exploring in more detail the relationship between cardosin A trafficking and plant development.

## 1 Introduction

Cardosins are well-characterized aspartic proteinases (APs) that were first isolated and characterized from cardoon flowers (Ramalho-Santos et al., 1998; Vieira et al., 2001). Cardosins A and B, two distinct but related proteinases, were identified and have been intensively researched in both native and heterologous systems throughout the years (Pissarra et al., 2007; Oliveira et al., 2010; da Costa et al., 2011; Pereira et al., 2013; Almeida et al., 2017). Although they have a high degree of similarity, they are products of different genes and show significant differences concerning enzymatic and kinetic properties (Ramalho-Santos et al., 1996). In general, cardosin B has a broader specificity than cardosin A, although it has higher activity. Based on this observation, it was proposed that while cardosin B may intervene in protein digestion in general, cardosin A would participate in more specific processes with a high degree of regulation (Ramalho-Santos et al., 1996). Later on, the genes coding for cyprosins and for cardosin C, D, and E, which have high similarity with cardosin A, were also isolated from *Cynara cardunculus* (Pimentel et al., 2007). Each of these enzymes is synthesized as a precursor and undergoes different cleavages along the endomembrane system in order to acquire its mature form, composed of heavy and light chains. Despite the high similarity between plant and animal APs, some specific characteristics of plant APs make them unique. Most of the plant APs described to date are synthesized as pre-proenzymes, or zymogens, and are subsequently converted into the mature form. The primary structure of the precursor molecules is similar to the plant APs, characterized by the presence of an N-terminal signal sequence responsible for addressing the protein to the endoplasmic reticulum, followed by a prosegment of approximately 40 amino acids, an N-terminal domain, and a C-terminal domain separated by a region of approximately 100 amino acids called a PSI (plant-specific insert) (Simões and Faro, 2004; Pissarra et al., 2007). The PSI has high homology with proteins similar to saposins (SAPLIPs), and every member of this family has six conserved cysteine residues forming three conserved disulfide bonds, a number of hydrophobic residues and, in some cases, a glycosylation site. The PSI has a particular characteristic when compared with saposins: there is a reversal of the N- and C-terminal domains of the saposins, where the C-terminal portion of the saposin is linked to the N-terminal. A scheme for processing cardosins has been proposed based on cardosin cleavage sites using specific antibodies for different regions of the protein (Ramalho-Santos et al., 1998).

Studies of cardosins in cardoon illustrate the plurality and complexity of its sorting mechanisms (Pereira et al., 2008; Oliveira et al., 2010). In the floral tissues, cardosin A is mostly detected in the protein storage vacuoles (PSVs) of the stigmatic papillae, whereas cardosin B is located in the extracellular matrix of both the stigma and style transmitting tissues. In cardoon flowers, lytic vacuoles (LVs) and PSVs coexist in the same cells of the pistil. In these cells, cardosin A, which makes up approximately 60% of the total protein content in cardoon flowers, is mostly accumulated in PSVs (Ramalho-Santos et al., 1997; Pissarra et al., 2007). In vegetative tissues, like leaves and shoots, cardosin A is barely detected and is found again in high amounts in seeds, accumulating in protein bodies (PBs) (Pereira et al., 2008). This is a clear example of specialization to meet the cells’ unique requirements (Pereira et al., 2008). An alternative route bypassing the Golgi was proposed based on observations in cardoon seed. In the seeds, cardosins were detected in PBs and not at the Golgi, indicating the possibility of a Golgi-independent delivery. Therefore, various routes can be proposed for cardosin A, depending on the cell/tissue/organ in which it is to be expressed, as the available data suggest a possible specialization of its trafficking pathways according to the type of cell and specific needs. It was observed that depending on the cell type and developmental stage, the same protein can be secreted to the apoplast, directed to the vacuole, or accumulated in PSVs or LVs traveling through different routes, implying a tight mechanism of trafficking regulation. The vacuolar pathways that some proteins take in specific organs and how those routes change in organs with varied metabolic activity have sparked new debate.

Initially, several possibilities for cardosin A functions were proposed: involvement in floral senescence and participation in programmed cell death; involvement in defense against pathogens; involvement in pollen–pistil interaction (Ramalho-Santos et al., 1997); and remodeling and permeabilization of membranes during germination (Pereira et al., 2008). Cardosin B has also been implicated in the phenomenon of programmed cell death in the nucellar layers of the egg (Figueiredo et al., 2006). In general, the expression of cardosin A seems related to organs and tissues that are undergoing substantial morphological changes, and its expression is most likely regulated during plant development (Faro et al., 1999; Pereira et al., 2008). Preprocardosin A is processed along the vacuolar pathway, with the last processing step being the removal of the internal PSI domain taking place in the vacuole, probably due to its acid environment (Duarte et al., 2008). However, in cardoon seeds, an unprocessed precursor form of cardosin A reaches the PB in a suggested Golgi-independent manner (Pereira et al., 2008). Furthermore, using antibodies against the PSI region, the precursor form of cardosin A was detected in the cell wall of cardoon seeds. Processed cardosin A is subsequently detected in the central LV as it engulfs PBs (Pereira et al., 2008). Using transient expression systems in *N. tabacum* leaves, it was possible to identify two vacuolar sorting determinants in cardosin A: the PSI and a C-terminal signal. It was demonstrated that each domain determines a different route to the vacuole in *N. tabacum* leaves: with the PSI as a vacuolar sorting determinant (VSD), cardosin A is able to bypass the Golgi to reach the vacuole, while with the C-terminal peptide, it follows a classic endoplasmic reticulum (ER)–Golgi–prevacuolar compartment (PVC) route to the vacuole (Pereira et al., 2013). In the experimental model used, the C-terminal signal is dominant over the PSI signal, as it dictates the route the intact protein takes to the vacuole. However, it is likely that the two signals act independently and provide the APs with functional plasticity, controlling vacuolar trafficking according to cell constraints or needs. We postulate that non-glycosylated PSI of cardosin A could mediate an alternative, COPII-independent, vacuolar route and that this PSI-mediated pathway may be more relevant in metabolically active organs (such as flowers and seeds), where PSVs are predominant over LVs and cells have different needs, offering, therefore, the cells functional plasticity.

Seeds are a model of choice for intracellular studies in plants, particularly concerning vacuolar sorting, given the massive reorganization occurring in a short period of time, which makes studying cellular and subcellular changes in Arabidopsis embryo and seed tissues particularly challenging. After dormancy, storage proteins and minerals need to be mobilized from the PSV, which eventually are converted into LVs during seed germination, but this mechanism remains a matter of debate (Shimada et al., 2003; Bolte et al., 2011; Feeney et al., 2018). Several studies have explained the complex vacuole organization and the remodeling of vacuolar membranes occurring during seed germination (Maeshima et al., 1994; Inoue et al., 1995; Olbrich et al., 2007; Bolte et al., 2011). However, while trafficking to the LV has been extensively reviewed in recent years, there is still no consensus regarding specific trafficking to the PSV, which remains the subject of significant research questions (Ashnest and Gendall, 2018).

The main objective of this work was to have a detailed view of cardosin A trafficking and accumulation in young seedlings, i.e., a few days after seed germination, where the vacuolar system experiences intense remodeling and different types of vacuoles co-exist. Immunocytochemical analysis of young Arabidopsis seedlings was performed using different antibodies, allowing the correlation of cardosin A processing events with its localization along the vacuolar pathway. Cardosin A labeling patterns were compared with well-known markers for pre-vacuolar compartments—m-Rab—and for PSV—TIPs—in germinating seedlings. A Brefeldin A (BFA) assay was also carried out to assess the cardosin A route in the cells of these seedlings. Results showed that cardosin A accumulates in PSVs during seed germination. Furthermore, cardosin A, at least in part, could reach the PSV, overcoming the BFA blockage and possibly bypassing the Golgi. These findings reinforce the hypothesis of a PSI A-mediated route that does not involve the Golgi; instead, it takes the protein directly to the vacuole using yet unknown mechanisms. The possibility that the PSI can act inside the protein is also discussed in light of the data presented here. The exact mapping of the cardosin A vacuolar pathway in young seedlings will retrieve interesting information about cardosin A involvement in membrane remodeling and mobilization of protein reserves, as proposed in the past (Pissarra et al., 2007; Pereira et al., 2008).

## 2 Materials and methods

### 2.1 Arabidopsis inducible system

Arabidopsis plants expressing cardosin A under a dexamethasone-inducible system (Samalova et al., 2005) were already available (Duarte et al., 2008) and had previously been selected by reporter expression up to the T5 generation. In this work, we selected individuals for the T6 generation and tested them for expression of the reporter and the transgene. Arabidopsis seedlings were sterilized in 70% (v/v) ethanol and transferred to MS medium containing 1.5% (w/v) sucrose and 0.7% (w/v) Bacto-agar. When working with non-homozygous plants, 20 µg/ml hygromycin was added to the culture medium to ensure selection of the resistant seedlings. After stratification, seeds were incubated at 21°C with a 12 h light photoperiod for 12–15 days and then transferred to individual pots with the fertilized substrate (SiroPlant) and maintained in a growth chamber with the same conditions. Induction of transgene expression can be accomplished either in liquid or in solid culture medium, with the conditions being the same for both cases. The induction of transgene expression was performed in MS medium with 20 µM dexamethasone for periods ranging from 24 h to 7 days at 21°C under a 12 h light photoperiod.

### 2.2 GUS staining of Arabidopsis plants

The screening of transgenic lines can be easily accomplished through GUS staining. This colorimetric assay allows the detection of the activity of the protein coded by the GUS reporter gene—β-glucuronidase. Arabidopsis seedlings were vacuum infiltrated with GUS staining solution [75 mM phosphate buffer (pH 7.0), 10 mM EDTA, and 0.5 mg/ml 5-bromo-4-chloro-3-indolyl-β-D-glucopyranoside (X-Gluc, stock solution in DMF, Sigma)], and incubated at 37°C for 12 h. After incubation, the staining solution was replaced with 100 µl of 70% (v/v) ethanol, and the samples were incubated at 42°C for 30 min. Seedlings were de-stained overnight in 70% (v/v) ethanol at room temperature.

### 2.3 Protein extraction and Western blotting

To test for the presence of the transgene, seeds were germinated in a solid medium and, after reaching the three-rosette leaf stage, placed in a liquid medium with the inducer agent dexamethasone for 3 days. The seedlings were then collected and frozen in liquid nitrogen before protein extraction. Proteins were extracted from leaf tissue in the following extraction buffer: two volumes of extraction buffer [50 mM sodium citrate, pH 5.5; 5% SDS (*w*/*v*); 0.01% BSA (*w*/*v*); 150 mM NaCl; 2% (*v*/*v*) β-mercaptoethanol; and 10 µl of protease inhibitor cocktail (Sigma-Aldrich)] per 300 mg of the tissue sample. After maceration, the tissue boiled for 10 min. The supernatant was collected after centrifugation of the samples at maximum speed for 30 min at 4°C. Protein samples were then subjected to an SDS-PAGE denaturing gel followed by Western blotting using the anti-cardosin A antibody (**Supplementary Table 1**). A 12% polyacrylamide gel SDS-PAGE was obtained using 5 µl of total protein extract (approximately 10 µg) per sample, and 5 µl of Page Ruler Plus Prestained (ThermoScientific) was the chosen protein molecular weight marker. Once electrophoresis was completed, the proteins were transferred to a nitrocellulose membrane with a Tris-glycine-methanol buffer. The following solution was used for a 1 h blockage of the membrane: Tris-buffered saline supplemented with 5% (*w*/*v*) skim milk, 1% (*w*/*v*) bovine serum albumin, and 0.6% (*v*/*v*) Tween 20. The membrane was probed using anti-cardosin A at 4°C, overnight. Alkaline phosphatase-conjugated secondary antibody (Vector) was used at a 1:1,000 dilution for 1 h at room temperature, and the proteins were exposed to Novex AP Chromogenic substrate (Invitrogen), according to the manufacturer’s protocol.

### 2.4 Arabidopsis growth stage-based phenotypic assay

A detailed assay based on development processes was carried out as described in Boyes et al. (2001) (Boyes et al., 2001), comparing wild-type plants with induced and non-induced plants expressing cardosin A. Seeds were germinated in solid medium, as described above, and were passed to the soil in the seedling stage when the root was more than 6 cm long. The induced plants were watered with a solution of dexamethasone. The time of different developmental points was recorded for further analysis: seed imbibition; radicle emergence, hypocotyl, and cotyledon emergence; four stages of leaf development—cotyledons fully opened and two, three, and four rosette leaves; first flower buds visible; first flower open; and presence of first siliques.

### 2.5 PEG embedding

Arabidopsis 3-day-old seedlings (72 h of germination) were selected for PEG embedding. The seedlings were fixated in 3% (v/v) paraformaldehyde diluted in 1× PBS (140 mM sodium chloride, 2.7 mM potassium chloride, 10 mM sodium hydrogen, and 1.8 mM potassium hydrogen) at room temperature for 90 min in glass vials, with constant shaking. After fixation, five washes were made in PBS 1×, for 10 min each. Then, the process of dehydration was initiated by incubating the samples in a graded ethanol series for 30 min each mixture—30% (v/v), 50% (v/v), 70% (v/v), 90% (v/v), and 100% (three times). The samples were maintained in the last 100% ethanol and placed at 42°C for 10 min to reach the embedding medium temperature. An equal volume of wax solution (90 g of PEG 400 distearate and 10 g of hexadecanol, mixed at 65°C) was added to the 100% ethanol bath and incubated overnight at 42°C. The mixture was then replaced by a pure wax solution and maintained at 42°C for at least 4 h. The specimens were placed in plastic embedding molds and left to solidify at room temperature. Fully polymerized blocks were conserved at 4°C.

### 2.6 Poly-L-lysine coating

The slides used for immunolocalization were coated with poly-L-lysine. New slides were washed with Extran MA 0.1 alkaline (Merck) for 5 min and rinsed in water to remove the excess detergent. The slides were incubated for 5 min in 70% (v/v) ethanol, then in 0.1% (v/v) poly-L-lysine solution for 10 min and left to dry at 55°C for 2–3 h.

### 2.7 Immunolocalization in PEG sections

Thin sections of PEG-embedded blocks were placed in poly-L-lysine-coated slides moistened with 1× PBS and allowed to dry on the slides overnight. The tissues were then rehydrated by a decreasing series of ethanol (100%, 100%, 100%, 90%, 70%, 50%, and 30%, prepared in PBS) for 10 min each. The slides were washed twice in 1× PBS for 10 min and incubated in a blocking solution (10 mg/ml BSA in 1× PBS) for 15 min. The incubation with the primary antibody diluted in 1× PBS (**Supplementary Table 1**) was performed in a moistened chamber at 26°C for 1 h, followed by 5 washing steps in 1× PBS with 1% (v/v) fish gelatin, with a duration of 10 min each. Sections were then incubated with the secondary antibody (Alexa 488®, Invitrogen), diluted in PBS, at room temperature for 1 h. Two new washes were made in 1× PBS with fish gelatin for 10 min, and the excess liquid was removed. The slides were coated with a few drops of Citifluor® and sealed with a coverslip and nail polish. The slides were stored at 4°C in the dark prior to imaging. Samples were imaged with a confocal laser scanning microscope (CLSM, Leica SP2). Alexa 488 emission was detected using 488 nm excitation, and fluorescence emission was detected between 500 and 528 nm. Transmission images were taken simultaneously in Nomarski DIC imaging mode. Image analysis was performed with the free software ImageJ/Fiji (https://imagej.net/software/fiji/).

### 2.8 BFA treatment assay

Arabidopsis seedlings were germinated in a liquid MS medium, and after 3 days (72 h) germinating, dexamethasone was added to the medium. A total of 24 h later (to allow initial cardosin A expression), 50 µg/ml BFA was added, and samples were collected at 0, 2, 4, 8, and 20 h of drug treatment. The seedlings were cryofixed for LR White and epoxy resin inclusion. As a control, non-induced seedlings were used and treated similarly.

### 2.9 Cryofixation and cryo-substitution for LR White resin inclusions

For cryofixation, the plant material (Arabidopsis radicles and cotyledons) was cut into small pieces and high-pressure frozen using the EMPACT2 system (Leica) as indicated by the manufacturer. The samples were kept in liquid nitrogen until further processing. The samples were subsequently freeze-substituted with the AFS2 system (Leica) using the program described in **Supplementary Table 2**.

### 2.10 Cryofixation and cryo-substitution for epoxy resin inclusions

For cryofixation, Arabidopsis radicles and cotyledons were cut into small pieces and high-pressure-frozen using the EMPACT2 system (Leica) as indicated by the manufacturer. The samples were kept in liquid nitrogen until further processing. The samples were subsequently freeze-substituted with the AFS2 system (Leica) using the program described in **Supplementary Table 2**. The specimens were then removed from the AFS2 system and placed on ice for further processing. The samples were washed three times in ice-cold acetone for 1 h each before embedding. For the embedding steps, a series of mixed baths were prepared where the solvent (propylene oxide) was progressively substituted with the Epoxy resin (Agar low viscosity premix kit medium, Agar) as follows: ¼ resin + ¾ propylene oxide for 1 h, ^1^/_3_ resin + ^2^/_3_ propylene oxide for 3 h, ½ resin + ½ propylene oxide for 16 h, ^2^/_3_ resin + ^1^/_3_ propylene oxide for 1 h, ¾ resin + ¼ propylene oxide for 3 h, pure resin for 1 h, pure resin for 16 h, pure resin for 8 h, and finally pure resin for 16 h. The polymerization was performed at 60 °C for 18 h.

### 2.11 Block sectioning

The sectioning of LR White and epoxy resin blocks was made in an Ultracut UC6 (Leica) ultramicrotome, and ultrathin sections of approximately 70–90 nm were obtained. The LR White sections were placed on 75-mesh carbon-formvar-covered nickel grids, and the Epoxy resin sections were placed on 300-mesh copper grids.

### 2.12 Immunogold labeling in LR White sections

Grids were incubated (with the sections facing down) in drops of 1× TBS (10 mM Tris and 130 mM NaCl, pH 7.4) with 1% (v/v) Tween 20 for 10 min, followed by an incubation of 10 min in 1× TBS with 100 mM glycine. Grids were then transferred to 1× TBS + 100 mM glycine drops with 0.25% (v/v) acetylated BSA for 10 min. The incubation with the primary antibody (**Supplementary Table 1**) was performed in drops of 1× TBS + 100 mM glycine + 0.25% acetylated BSA for 1 h. After incubation with the primary antibody, five washes were made in 1× TBS with 0.25% acetylated BSA, with a duration of 5 min each. The incubation with the secondary antibody coupled to 15 nm or 10 nm colloidal gold (Agar Scientific; 1:20 dilution) was performed in 1× TBS + 100 mM glycine for 1 h. Five washes in 1× TBS of 5 min each were undertaken, followed by five more washes in distilled water of 5 min each. The grids were then allowed to dry on filter paper.

### 2.13 TEM observations

Grids containing ultrathin sections (both from LR White or epoxy) were post-stained with an aqueous solution of 2% (w/v) uranyl acetate/lead citrate. For the contrasting step, a 2% (w/v) uranyl acetate solution (15 min incubation) was used, followed by five washes in distilled water. The incubation in lead citrate (5 min incubation) was performed in a Petri dish with KOH or NaOH pellets to have a CO_2_-free atmosphere and was followed by five washes in distilled water. Grids were allowed to dry on filter paper and stored. Grids were then examined under a JEOL 1400 TEM operating at 120 kV. Images were acquired using a post-column high-resolution (11 megapixels) high-speed camera (SC1000 Orius, Gatan). Image analysis was carried out using the free software ImageJ/Fiji (https://imagej.net/software/fiji/). Quantification of cardosin A after BFA treatments were conducted using acquired micrographs, and gold dots inside PSVs were counted at all time points and plotted in a bar graph. An ordinary one-way ANOVA using Tukey’s multiple comparison test was used to compare all the situations (*n* = 9, for each condition). Analysis was done using GraphPad Prism 8.0.1.

## 3 Results

Cardosins sorting to the vacuole have been studied and characterized in *N. tabacum* leaf epidermis, resorting to Agrobacterium-mediated transient expression. Despite a large amount of information gathered over the years, this system has limitations in fully understanding cardosins’ sorting. Therefore, to fully explore cardosins’ trafficking and validate their proper expression in the Arabidopsis system, studies on cardosin A expression and trafficking have been extended to Arabidopsis. Young seedlings are a model of choice for intracellular studies in plants, particularly concerning vacuolar sorting, given the vast changes occurring in a short time. We chose to study cardosin A accumulation and trafficking in germinating Arabidopsis seedlings to further understand cardosins’ trafficking in plant cells.

### 3.1 Cardosin A inducible system

Along with the existing constitutive expression commonly used for stable Arabidopsis transformation and keeping in mind the need to control the induction of cardosin A expression, we chose an inducible expression system for the study of cardosin A biogenesis and localization. Samalova and coworkers (2005) (Samalova et al., 2005) developed an inducible expression system—LhGR— where the occurrence of a phenotype with the expression of a transgene only occurs upon induction with a chemical agent—dexamethasone (Dex). Arabidopsis lines expressing cardosin A under the LhGR d examethasone-inducible promoter were already available (Duarte et al., 2008), and cardosin A expression was partially characterized in vegetative tissues.

**Figure 1:**
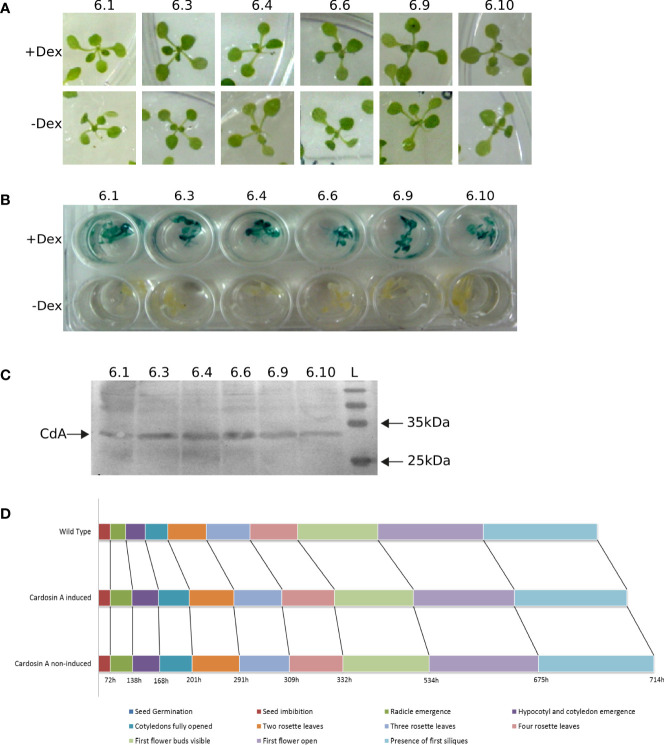
Characterization of Arabidopsis lines expressing cardosin A under an inducible promoter. **(A)** Germination and growth of T6 generation individuals in MS medium supplemented in the presence of dexamethasone and hygromycin. **(B)** GUS activity assay in induced (+ Dex) and non-induced (− Dex) seedlings. **(C)** Western blot of leaf protein extracts from Arabidopsis lines expressing cardosin A, detected with an anti-cardosin A antibody. **(D)** Schematic of the chronological progression of the 10 principal growth stages chosen for phenotypic comparison between Arabidopsis cardosin A lines and wild-type plants. More than 20 plants were observed with similar results in two independent experiments. Dex, dexamethasone; L, all blue prestained protein ladders (BioRad).

To fully interpret any data on the dynamics and localization of cardosin A in these plants, we checked for the expression of the protein over several generations, the correct processing of cardosin A, and the integrity of the ultrastructural architecture of the cells. Six independent T6 lines germinated in dexamethasone and hygromycin (**Figure 1A**), and all the individuals were resistant to the antibiotic, confirming that every line was indeed homozygous. Furthermore, each plant was tested independently for the expression of the reporter gene (GUS) and the transgene (cardosin A). A GUS activity assay was carried out in dexamethasone-induced and non-induced plants to serve as a control for the leaky expression of the reporter. The six lines tested showed a good expression of the reporter (+ Dex plants), and no expression was detected in the non-induced plants (− Dex) (**Figure 1B**). Finally, the confirmation of cardosin A expression and correct processing was carried out by Western blotting using protein extracts obtained from the leaves of dexamethasone-induced plants and a specific antibody against cardosin A. The results showed a clear band of approximately 31 kDa corresponding to the heavy chain of the protein (mature form), confirming that cardosin A is correctly processed in this system (**Figure 1C**).

There was no apparent phenotype in the transformed plants when compared to wild-type (WT) plants or non-induced ones. Nevertheless, a more detailed assay was carried out to confirm the progression of the main growth stages during Arabidopsis development (Hunter et al., 2007). A total of 10 different developmental stages were monitored: seed imbibition; radicle emergence; hypocotyl and cotyledon emergence; four stages of leaf development: cotyledons fully opened and two, three, and four rosette leaves; first flower buds visible; first flower open; and presence of first siliques. The duration of each developmental stage was monitored in more than 20 plants for each condition (induced, non-induced, and WT) in two independent experiments. The results obtained from the developmental observations are schematically represented in the chronological progression for each condition in **Figure 1D**. A slight delay in cardosin A (both induced and non-induced) seed germination is visible when compared to the WT control. This slight delay was observed throughout development.

**Figure 2:**
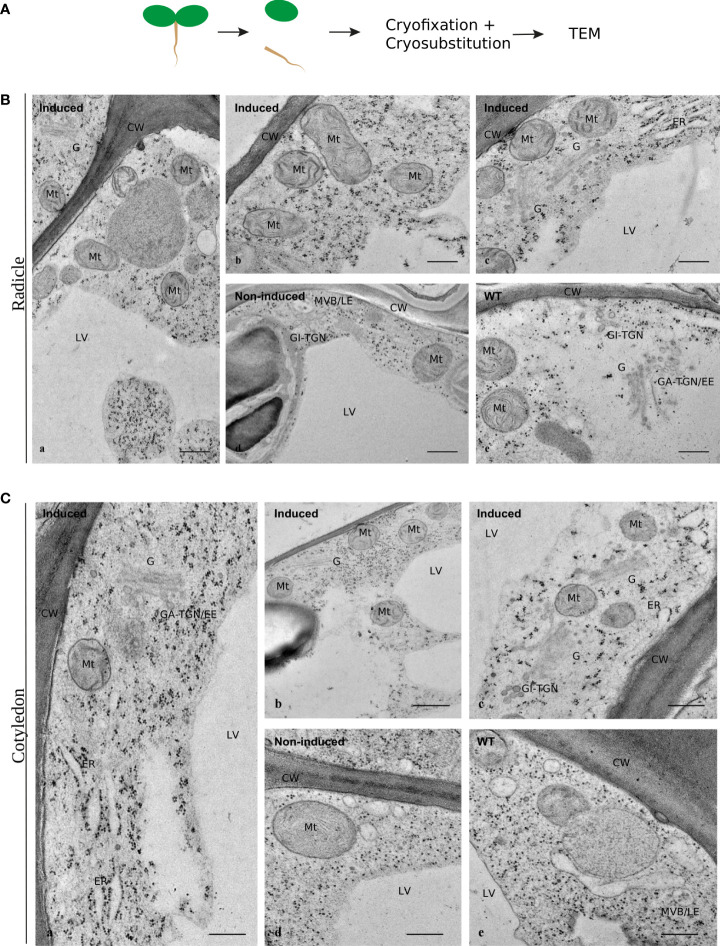
Ultrastructural aspects of Arabidopsis cells expressing cardosin A under a dexamethasone-inducible promoter. Micrographs of cell ultrastructure were obtained from 3-day-old seedlings, cryofixed, and cryo-substituted before embedding in epoxy resin. **(A)** Sections of radicles evidencing the general organization of the cells in induced plants (a), and details of several well-preserved organelles (b, c). Non-induced (d) and wild-type (e) seedlings were used as controls. Scale bars: a, 500 nm; b–e, 250 nm. **(B)** Sections of cotyledons evidencing the general organization of the cells (a, b) and details of several intact organelles **(C)**. As a control, 3-day-old non-induced (d) and wild-type (e) seedlings were used. Scale bars: a, c, 400 nm; b, 500 nm; d, e, 300 nm. CW, cell wall; LV, lytic vacuole; ER, endoplasmic reticulum; G, Golgi body; GA-TGN, Golgi-associated TGN; GI-TGN/EE, Golgi independent TGN/early endosome (also named free TGN or late TGN); MVB/LE, multivesicular body/late endosome (or PVC).

Finally, Arabidopsis wild-type plants and cardosin A-expressing lines were analyzed at the subcellular level to ascertain if there were phenotypic differences in their ultrastructure. Radicles and cotyledons were excised from 3-day-old seedlings and analyzed separately by TEM. Radicle cell (**Figure 2A**) structure was comparable between induced (**Figure 2A**, b, c), non-induced (**Figure 2A**, d), and wild**-**type (**Figure 2A**, e) plants. The morphology, shape, and overall size of several subcellular structures, namely, Golgi bodies and mitochondria, were similar. Likewise, in cotyledon cells (**Figure 2B**), the ultrastructure revealed no major subcellular effect of cardosin A expression in cells or organelles. Nevertheless, we noticed dilated ER cisternae in a few micrographs (**Figures 2B**, c, **C**, a, c), which could be related to overexpression of cardosin A. Golgi stacks were abundant in both radicle and cotyledon cells associated with the *trans*-Golgi network (GA-TGN) (**Figure 2C**, a), and free Golgi-independent TGN (GI-TGN/late TGN) (**Figure 2C**, c) was also observed. These morphological observations seem to indicate that cardosin A expression did not cause any major disturbance of the plant endomembrane system, and consequently, the secretory, endocytic, and vacuolar pathways are potentially fully functional in these cells.

### 3.2 Cardosin A localization in seedlings depends on the type of tissue

**Figure 3:**
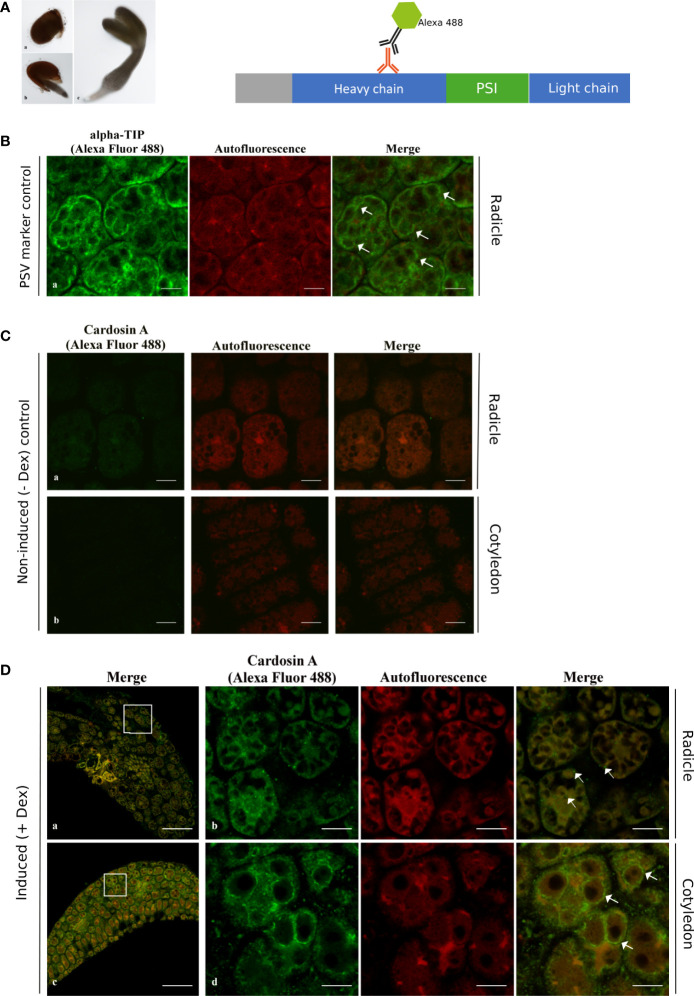
Immunofluorescence of cardosin A in radicle and cotyledon sections of the Arabidopsis inducible expression system using a polyclonal antibody against cardosin. Seeds were germinated in the presence of dexamethasone and 3 days after germination, seeds were fixed, embedded in PEG, and analyzed by confocal laser scanning microscopy. **(A)** Schematic representation of procedure. **(B)** Immunodetection using anti-α-TIP with Alexa fluor 488® secondary antibody as a PSV marker. **(C)** Immunolabeling of non-induced plants. **(D)** Cardosin A labeling in the radicle appears as punctate dots; labeling in the radicle sections in the cytoplasm (a and b, merged white arrows) and in the cotyledons is mostly detected surrounding the PSVs (c, d, merged white arrows). Scale bars: **(B)** 9 µm, **(C)** 9 µm, **(D)** a, c 45 µm, and b, d 9 µm.

As a starting point, 24 h and 72 h after germination time points were analyzed, but only immunofluorescent labeling was obtained for the 24-h time point, and although the results for the radicle were inconclusive in order to discern localization (**Figure S1B**, a-c), the results for the analyzed cotyledons were very similar to the results 72 h (3-day-old) post-germination (**Figure S1B**, d-f). From this point forward, we are referring to the 3-day-old post-germination only. Two different plant tissues from young seedlings were analyzed 3 days after germination (**Figure 3A**, c).

Cardosin A localization was first analyzed by immunofluorescence in radicle and cotyledon sections using a polyclonal anti-cardosin A antibody and a secondary antibody coupled to Alexa Fluor 488®. At the same time, autofluorescence images were acquired to highlight the tissues’ intrinsic autofluorescence. Cardosin A labeling was detected in radicles and cotyledons in practically all cell types (**Figure 3D**, a, c), which is not surprising given the usage of an LhGR promoter in the construction of the binary vector. However, when zooming in on the cells, it appears that intracellular labeling differs between radicles and cotyledons (**Figure 3D**, b, d). In radicle cells, cardosin A was found in dots in the cytoplasm (**Figure 3D**, b merge, white arrows) and the cotyledons, and it was also detected surrounding the PSVs (**Figure 3D**, d merge, white arrows). PSVs are identifiable by the characteristic red autofluorescence [described in (Bolte et al., 2011)], and immunolabeling with anti-α-TIP is also presented (**Figure 3B**), and this is very similar to cardosin A localization surrounding PSVs. α-TIP is known as a specific marker for vacuoles containing seed storage proteins—PSVs (Hunter et al., 2007). Immunofluorescence for α-TIP was performed in radicle sections of 3-day-old Arabidopsis seedlings expressing cardosin A under a dexamethasone-inducible promoter. No labeling was detected in either the radicle or cotyledons of non-induced plants (**Figure 3C**). This first set of data shows a differential localization of cardosin A depending on the tissue, i.e., radicle compared to cotyledons.

**Figure 4:**
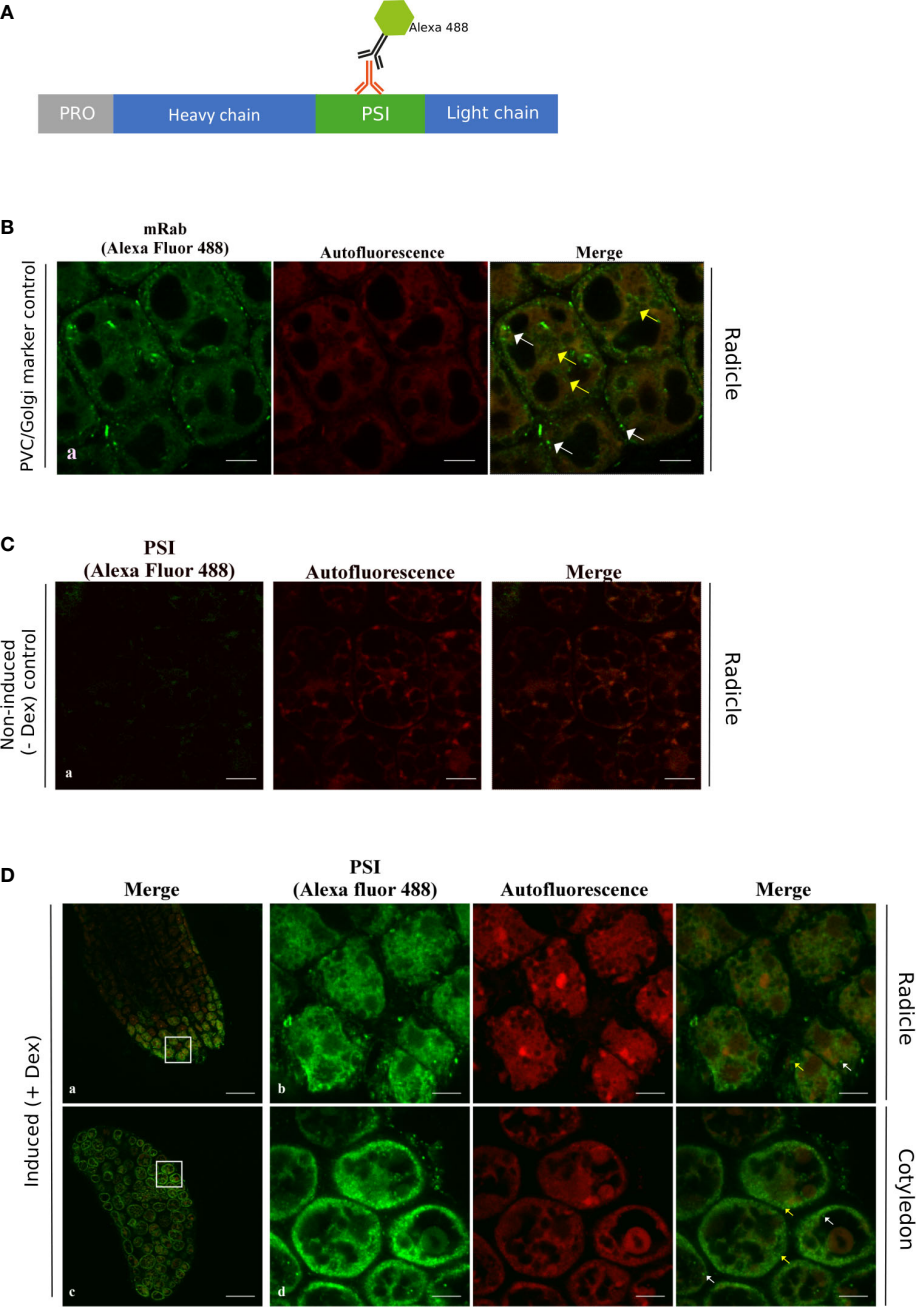
Immunofluorescence using the anti-PSI antibody in radicle and cotyledon sections of the Arabidopsis inducible expression system. Seeds were germinated in the presence of dexamethasone and fixed, embedded in PEG, and analyzed by confocal laser scanning microscopy3 days after imbibition. **(A)** Schematic representation of the procedure. **(B)** Immunodetection using anti-mRab with Alexa fluor 488® secondary antibody as a PVC marker. **(C)** Immunolabeling of non-induced plants. **(D)** PSI labeling was found with small vesicles in the cytoplasm as in the control (yellow arrows) and was also detected in dot-shaped compartments in the vicinity of the lytic vacuole, which is also similar to the mRab control (white arrows). Scale bars: **(B)** 7 µm, **(C)** 9 µm, and **(D)** a, c 45 µm, and b, d 9 µm.

Using an antibody recognizing the cardosin A-PSI region, it was possible to relate cardosin A processing events with cardosin A localization along the vacuolar pathway (**Figure 4A**). This antibody allowed the recognition of the precursor form of cardosin A; the PSI region was still attached to the light chain and the PSI alone and would not detect the processed heavy chain as the antibody to cardosin A does. As for cardosin A, we also observed the 24-h time point, and the results were very similar to cardosin A (**Figure S1**); therefore, we carried on only with the 72-h (3-day-old) samples. In radicle sections of 3-day-old seedlings, the labeling of the anti-PSI antibody was similar to the one obtained with the cardosin A antibody, as it appears in small vesicles in the cytoplasm (**Figure 4D, a, b** yellow arrows). In cotyledon sections of the same germination stage (3-day post-germination), the localization is different from cardosin A as in the anti-PSI-labeled structures, and the fluorescence appears in dot-shaped compartments throughout the cytoplasm of the cells (**Figure 4D**, d yellow arrows) and a few dot-shaped compartments in close proximity or possibly inside the LV (**Figure 4D**, d merge white arrows). The detection pattern is similar to the pattern detected for the anti-mRab, which was found to localize at the PVC and partially in association with the Golgi apparatus (Bolte et al., 2004) (**Figure 4B**). Immunofluorescence for mRab was performed in radicle sections of 3-day-old Arabidopsis seedlings expressing cardosin A under a dexamethasone-inducible promoter. No labeling was detected in either the radicle or cotyledons of non-induced plants (**Figure 4C**). Comparing the results obtained with anti-cardosin A and anti-PSI, it can be seen that the fluorescence pattern obtained with the two antibodies is different. This is an indication that the cardosin A detected in association with the PSVs (using an anti-cardosin A antibody) may be the processed one without the PSI domain. The PSI detection showed a punctate pattern in the cytoplasm, suggesting that these could be precursor forms in transit along the secretory pathway, but the mature form detected by the anti-cardosin A was more restricted to the PSVs, indicating the presence of the mature form in the PSVs. These results give an insight into the processing of cardosin A along the pathway to the PSV and suggest that the PSI domain may be cleaved, but do not allow us to conclude exactly when along the pathway of the PSI domain is cleavage occurring in cotyledons. Given the detection of the PSI domain in proximity with the LV, it would be important to characterize its localization throughout the entire vacuolar system and therefore distinguish other types of vacuoles. Transmission electron microscopy and immunocytochemistry were performed to help answer these questions.

### 3.3 Immunoelectron microscopy of cardosin A at the ultrastructural level reveals its association with a complex vacuolar system

**Figure 5:**
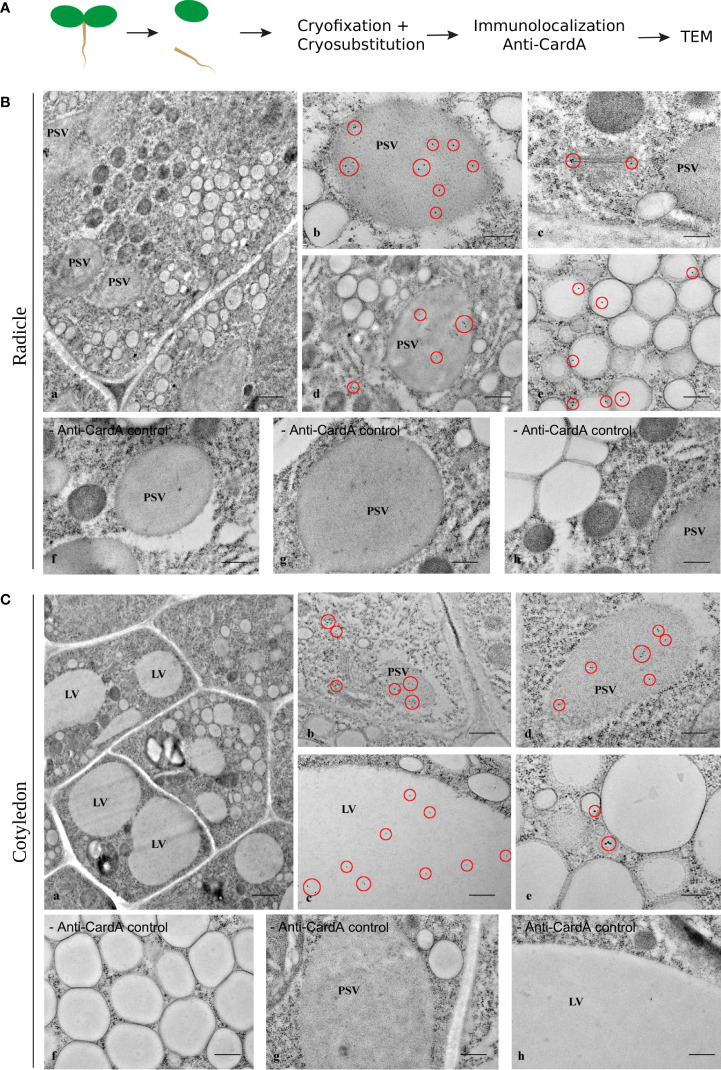
Immunogold labeling of cardosin A in the Arabidopsis inducible expression system. Three-day-old seedlings, germinated in the presence of dexamethasone, were cryofixed and embedded in LR White resin for immunoelectron microscopy. **(A)** Schematic representation of the procedure. **(B)** Radicle sections: (a) General view of the cells; labeling was detected inside protein storage vacuoles (b, d) and associated with the Golgi apparatus (c). Cardosin A was also detected in small, electron-transparent structures in the cytosol (e). The red circles mark the gold labeling corresponding to cardosin **(A)** As control of the immunolabeling technique, sections without primary antibodies (− anti-card A f-h) were used. Scale bars: a, 1 µm; b, c, g, h, 200 nm; d, f, 500 nm; e, 300 nm. **(C)** Cotyledon sections: (a) General view of the cells; labeling was detected inside protein storage vacuoles (b, d) and in the large central vacuole (c). Cardosin A was also detected in small, electron-dense structures in the cytosol (e). The red circles mark the gold labeling corresponding to cardosin **(A)** As control of the immunolabeling technique, sections without primary antibodies (− anti-cardA f-h) were used. Scale bars: a, 1 µm; b, 500 nm; c, f-h, 400 nm; d, 300 nm; e, 200 nm.

To gain a clear picture of cardosin and the PSI domain distribution, the information provided by immunofluorescence analysis was complemented at the ultrastructural level by transmission electron microscopy (TEM). Immunoelectron microscopy was performed using the same specific polyclonal antibody raised against cardosin A. As before, radicles and cotyledons were treated separately. In radicle sections, the cells are characterized by a complex vacuolar system, characterized by the presence of different types of vacuoles (**Figure 5A**, a). The PSVs are recognizable not only by their size and shape but also by their proteinaceous matrix, which is electron-dense in the micrographs. In the cytoplasm, electron-dense and electron-transparent vesicles are found in large numbers. The majority of cardosin A labeling was found in PSVs (**Figure 5B**, b, d, red circles), but it was also found in association with Golgi and ER (**Figure 5B**, c, d, red circles). Some gold dots were also detected in association with some electro-transparent compartments in the cytoplasm, presumably small LVs (**Figure 5A**, e, red circles). Compared with the immunofluorescence data and considering the labeling intensities observed at the TEM level, it is tempting to assume that most of the labeling observed at the light microscopy level would correspond to the PSV compartments. However, although the fluorescence in the radicle, seems to be localized in the cytoplasm at the light microscopy level, it is possible that it is also present within the PSVs but was not detected. No labeling was detected in the control section without the primary antibody (**Figure 5B**, f-h).

In cotyledon cells, the vacuolar system appears differently structured from radicle cells, with one or more large vacuoles occupying the cell (**Figure 5C**, a). The PSVs are still present in these cells but are smaller than in the radicle cells. Electron-dense and electron-transparent membranous compartments are still observed in the cytoplasm. Cardosin A localization is also different between cotyledon and radicle cells, as already observed in the immunofluorescence studies. In cotyledon cells, cardosin A is detected in the central LVs (**Figure 5C**, c) and also in the PSVs (**Figure 5C**, b, d). Cardosin A was also found in the periphery of the ER and the Golgi stacks (**Figure 5C**, b). The results obtained in cotyledon cells by immunoelectron microscopy are, in some way, different from the ones obtained with immunofluorescence techniques. In the cotyledon sections observed by immunofluorescence, no cardosin A labeling within LVs was visible. This may be due to the low-level expression of the protein, which is hard to detect at the light microscopy level. Taking together the results obtained from the two approaches, it is clear that cardosin A accumulates in and around PSVs and is also found in association with the ER and the Golgi. Cardosin A is also detected in the central LVs of this tissue (**Figure 5C**, c).

**Figure 6:**
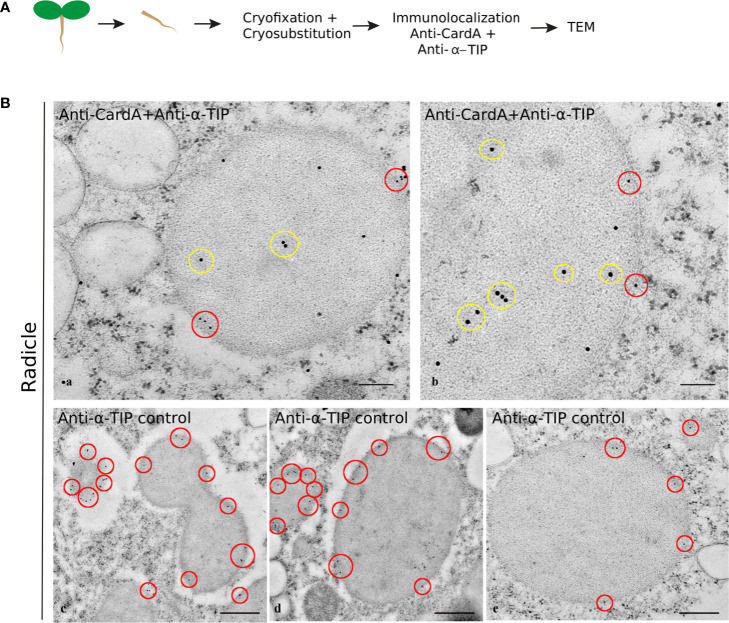
Double labeling of cardosin A and α-TIP in radicle sections of Arabidopsis expressing cardosin A under an inducible expression system. Specific antibodies against cardosin A and α-TIP were used coupled to a 15-nm (yellow circles) and 10-nm gold-conjugated secondary antibody (red circles), respectively. **(A)** Schematic representation of the procedure. **(B)** Cardosin A labeling was detected inside the protein storage vacuoles (a, b yellow circles), while anti-α-TIP conjugated with 10 nm gold particles decorates the periphery of the same structures (red circles). Anti-α-TIP conjugated alone was used as a control (c-e). Scale bars: **(B)** a, 300 nm; b, 200 nm; c, d, 500 nm; e, 200 nm.

In order to understand the identity of the cardosin A-labeled compartments, we compared the cardosin A labeling pattern with known markers for PVCs, PSVs, and LVs. As for immunofluorescence already shown, immunogold labeling was performed in radicle sections of 3-day-old Arabidopsis seedlings expressing cardosin A under a dexamethasone-inducible promoter. A double immunogold labeling of cardosin A and α-TIP was obtained to show the intracellular localization of the two proteins simultaneously. This technique allowed for a nice picture illustrating cardosin A accumulation in PSVs in radicle cells of Arabidopsis. Cardosin was detected in PSVs (15 nm gold dots), while α-TIP (10 nm gold dots, red circles) was found decorating the membrane (**Figure 6**, a, b). α-TIP is also presented alone (**Figure 6B**, c-e) as a control and shows similar accumulation on the PSV as for the double labeling. Another control was obtained, anti-γ-TIP, in order to identify LVs, but a dual localization was obtained (**Supplementary Figure S2**) since it is also described to label the PSV in seed tissues (particularly during seed germination and maturation) (Jiang et al., 2002; Hunter et al., 2007). Given the results obtained, it seems that these small electron-transparent round organelles may correspond to small LVs, possibly derived from MVBs that may eventually fuse to form a large central vacuole, as described by Bolte and their team (Bolte et al., 2011).

**Figure 7:**
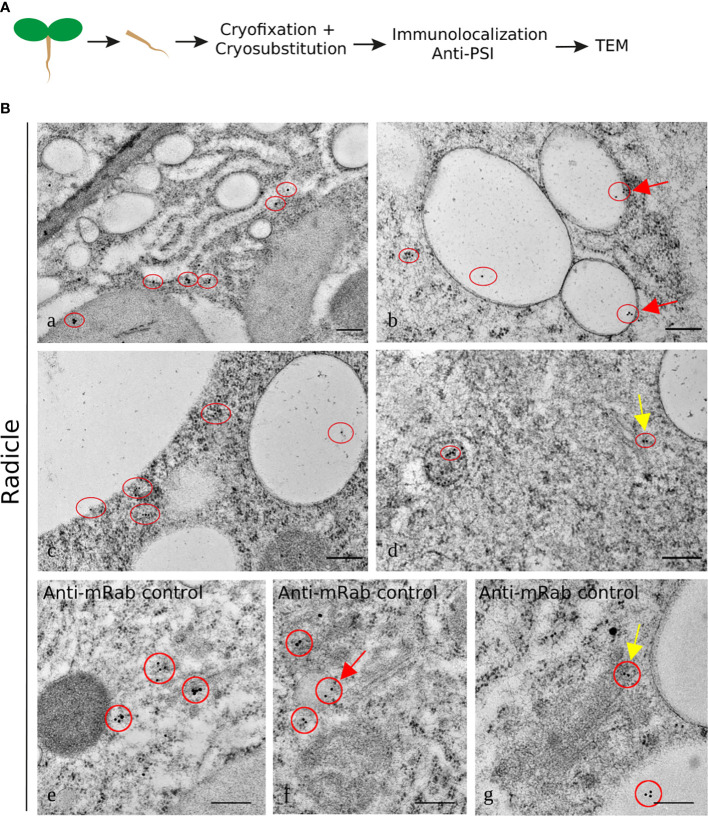
Immunogold labeling of PSI in the Arabidopsis inducible expression system. A specific antibody against PSI was used for immunolocalization in radicle cells. **(A)** Schematic representation of the procedure. **(B)** Detail of endoplasmic reticulum labeled with anti-PSI (a); labeling was detected in vacuoles, which is also observed in the control with mRab coupled to a 10-nm gold-conjugated secondary antibody (red arrows b, c, and f) and in association with a compartment that could be the multivesicular body (d) and also associated with the Golgi as for the control (d and g yellow arrows). The red circles mark the gold labeling corresponding to PSI (a–d) or mRab (e–g). Scale bars: a, 250 nm; b-d, 200 nm; e, f, 300 nm; g, 200 nm.

The results for the labeling using the anti-PSI antibody showed an association with the ER (**Figure 7A**) and with electron-transparent compartments, probably small vacuoles (**Figures 7B, C**). It is interesting to note that the labeling always appears near the membrane and rarely inside the organelle (red circles). This could indicate an association of the protein with membranes. The PSI was also detected in the vicinity of the LV (**Figure 7C**) and associated with the Golgi and organelles resembling PVC (**Figure 7D**). The detection of mRab was also presented, being very similar to the PSI detection in terms of Golgi association (**Figure 7**, yellow arrows). It was also detected in organelles that seem to be vacuolar, possibly LVs. As a whole, it is possible to say that cardosin A is detected along the secretory pathway and that it finally accumulates in the vacuole, PSV, or LV, depending on the tissue under study. These results are in accordance with previous results obtained in cardoon (Pereira et al., 2008).

### 3.4 Cardosin A can reach the PSV in the presence of BFA

**Figure 8:**
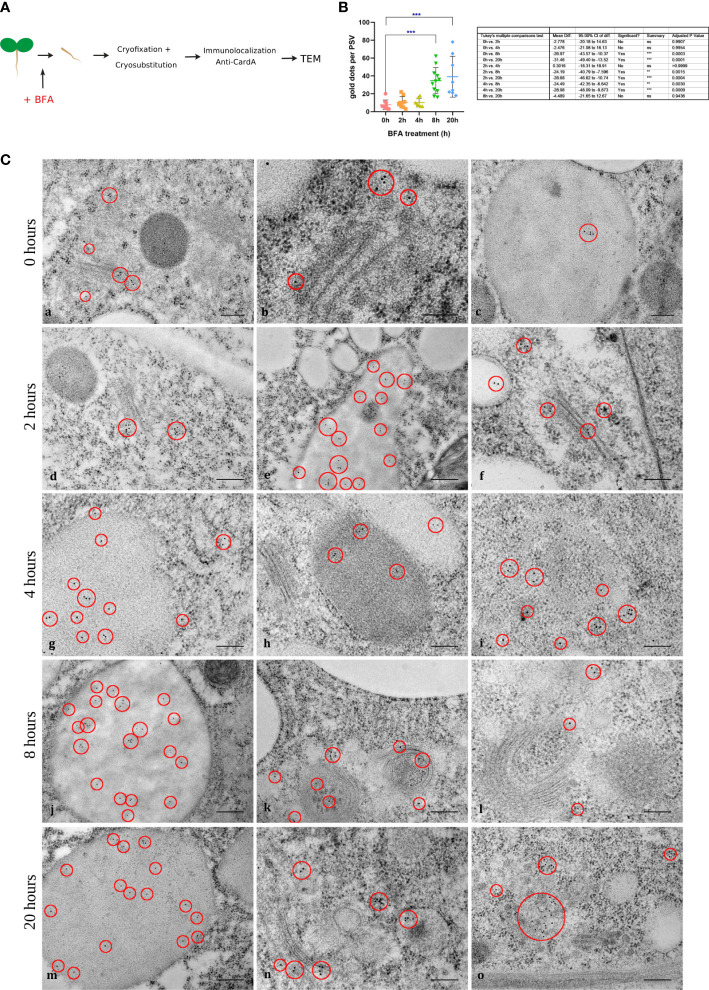
Immunogold labeling of cardosin A in radicle sections of the Arabidopsis inducible expression system at 0, 2, 4, 8, and 20 h upon addition of BFA. **(A)** Schematic representation of the procedure. **(B)** The exact time of BFA addition to the medium (0 h) was used as control (a-c). Cardosin A labeling was detected in association with the Golgi apparatus, with the protein storage vacuole, and also in the horseshoe-shaped Golgi bodies. This labeling pattern was maintained along the time points studied with a recognizable increase in Golgi bodies and TGN disassembly and bending to a horseshoe shape. Red circles mark the gold labeling of cardosin **(A)** Scale bars: a and c, 200 nm; b, 100 nm; d–l, 200 nm; m–o, 100 nm. **(C)** Quantification of cardosin A labeling inside PSVs, in radicle sections from immunogold labeling at 0, 2, 4, 8, and 20 h upon addition of BFA. Gold dots corresponding to cardosin A labeling inside PSVs was counted at all time points and plotted in a bar graph. An ordinary one-way ANOVA using Tukey’s multiple comparison test was used to compare all the situations (*n* = 9, for each condition). Analysis was done using GraphPad Prism 8.0.1. Statistically experimental values are represented by ***( pvalue ≤ 0.001).

At this point, a putative model for cardosin A biogenesis and trafficking in these tissues could be proposed, where cardosin A, synthesized in the ER, would travel through the Golgi and PVC towards the PSV. To validate this model, we started by blocking protein trafficking between cellular compartments with BFA and evaluating if cardosin A trafficking and/or localization were altered by this metabolite. The inducible line is useful here as it is possible to control the moment of cardosin A expression with the addition of the chemical inducer and coordinate that with the addition of BFA. BFA was added to 3-day-old seedlings 24 h after induction to allow the initial expression of cardosin A. Samples were collected at 0, 2, 4, 8, and 20 h upon addition of the drug and embedded in either epoxy or LR White resins for TEM analysis. Epoxy-embedded samples were used as a control to check for the structural effect of BFA treatment (**Supplementary Figure S3**). In the control sections (0 h BFA treatment), the Golgi and GA-TGN presented their recognizable stacked flattened cisternae shape, and indicators of polarity were easily discernible (*cis* to *trans* cisternae) (**Figure 8B**, a-c, **Supplementary Figure S3**). After 2 h and up to 20 h, BFA-treated samples showed disassembly of Golgi bodies and unstacking of cisterna; Golgi polarity was not recognizable, and Golgi cisternae became highly vesiculated, some of which could be ER–Golgi hybrid compartments as they became more electron-dense (**Supplementary Figure S3**). Several horseshoe-shaped Golgi bodies were also found (**Figure 8B**, j-l, **Supplementary Figure S3**). The other cellular organelles did not seem to be affected by the drug. Immunolocalization of cardosin A in BFA-treated tissues was performed at the same time points as for the ultrastructural studies to check the BFA effect. At the beginning of the study, corresponding to the control (0 h upon BFA addition), cardosin A labeling was found in the cytoplasm associated with the Golgi and with the ER (**Figure 8B**, a-c). A few gold dots were also detected within PSVs (**Figure 8B, c)**, indicating that, in this system, cardosin A trafficking to the vacuole is fast, since the production of cardosin A started just 24 h before. Cardosin A localization did not change during the time points studied: 2 h (**Figure 8B**, d-f), 4 h (**Figure 8B**, g-i), 8 h (**Figure 8**, j-l), and 20 h (**Figure 8**, m-o). Cardosin A is often found in association with the Golgi stacks or their close proximity (**Figure 8B**, d, f), even when the stacks have lost their regular shape (**Figure 8B**, k, n). Cardosin A accumulation in PSVs is also constant throughout the study (**Figure 8B**, e, g, h, j, and m, red circles); however, more gold dots could be detected in each PSV in the late time points—8 and 20 h—as confirmed by the quantification of cardosin A labeling (**Figure 8C**). This indicates that cardosin A may still be accumulating in the PSV despite the BFA blockage. Nevertheless, cardosin A accumulation in the Golgi apparatus indicates a route passing through this compartment in emerging radicles. This could corroborate previous observations in *N. tabacum*, indicating two possible pathways, one blocked by BFA treatment, the other unaffected (Duarte et al., 2008; Pereira et al., 2013). At the same time as the BFA treatment, a solvent-only (DMSO) control was obtained for each time point that showed no effect of the solvent (**Supplementary Figure S4**) as there was no disassembly of the Golgi stacks observed, and cardosin A localization was also similar to what was observed in **Figure 5**, previously characterized in this work. Localization using anti-PSI antibodies in BFA-treated cells was also obtained. PSI labeling was detected in association with the same compartments as for cardosin A BFA-treated cells, but it seems that there is less detection of PSI (red circles), which does not allow for further conclusions on its pathway and final accumulation. Nevertheless, we observed the same horseshoe Golgi bodies toward the prolonged exposure completely surrounding other organelles, which indicates that the Golgi bodies’ cisternal rims may have fused and this compartment could also be an ER–Golgi hybrid at this point. PSI was also detected in association with vesicles closely associated with ER and in proximity to Golgi bodies, which could be ER-derived carriers (**Supplementary Figure S5**), but further studies are needed to fully interpret these data.

## 4 Discussion

A crucial field of physiology, agriculture, and biotechnology research is the processing and subcellular trafficking of seed storage proteins. It has been considerably debated how to direct proteins to the LV. Trafficking to the LV has received a lot of attention in the past decades, but the routes to the LV compared to the routes to the PSV have not been scrutinized. Despite the considerable overlap between these pathways, the PSV has multiple instances of distinct processing and machinery (Ashnest and Gendall, 2018). The PSV is unique to plants, unlike the LV, which resembles yeast and animal vacuoles and lysosomes. Thanks to its sophisticated ultrastructure, the PSV can carry out a specialized storage role in addition to conventional vacuolar detoxification, protein turnover, and defense operations (Xiang et al., 2013). The seed storage proteins (SSPs), which accumulate in the PSV throughout seed development, are an essential product for agriculture and biology. However, a crucial research challenge is still how soluble enzymes are transported to the PSV at germination. Cardosins are well-characterized aspartic proteinases (APs) that were first isolated and characterized from cardoon flowers (Ramalho-Santos et al., 1998). Cardosins A and B have been extensively studied and characterized, revealing a high degree of regulation (Pissarra et al., 2007; Pereira et al., 2008). Cardosin A and cardosin B are located in different cellular compartments in flowers: cardosin A is in the vacuole, and cardosin B is secreted into the extracellular matrix (Ramalho-Santos et al., 1997; Vieira et al., 2001). Despite having a high degree of similarity in terms of nucleotide and amino acid sequences, this feature makes their study relevant. Cardosin A, in fact, accumulates in several types of vacuoles, depending on the organ studies, and data from both the native system (*Cynara cardunculus*) and transient expression in *N. tabacum* leaves indicate that several pathways exist depending on the requirements of the cell (Ramalho-Santos et al., 1997; Pereira et al., 2008; Pereira et al., 2013). Additionally, the PSI and the C-terminal peptide, two vacuolar sorting domains that relate to various routes to the plant vacuole, are present in both proteins. Therefore, these two proteins are superb tools for investigating vacuolar sorting and trafficking to the LV and PSV. Despite being a good system to explore cardosins’ sorting to the vacuole, transient expression in *N. tabacum* leaf epidermis does not answer the question regarding the different sorting of cardosins according to different tissues. In cardoon, cardosins are mostly expressed during flower and seed development (Ramalho-Santos et al., 1997; Vieira et al., 2001; Duarte et al., 2006; Figueiredo et al., 2006; Pereira et al., 2008), being only detected in residual amounts in vegetative tissues such as leaves or callus tissue (Oliveira et al., 2010).

### 4.1 Cardosin A accumulates in the PSV during germination

To fully explore cardosins’ trafficking and validate their expression in a stable expression system, studies on cardosin A expression and trafficking were extended to Arabidopsis seed cells. The main goal of this study was to have an integrated view of how/if the type of tissue in plants may affect protein localization. The “flexibility” of cardosin A trafficking pathways and their adaptation according to plant cell needs were studied 3 days after seed germination. The exact mapping of cardosin pathways in young seedlings and their multiple types of vacuoles adds important additional clues about their possible involvement in membrane remodeling and mobilization of protein reserves, as proposed in the past (Pereira et al., 2008). Arabidopsis lines expressing cardosin A were already available for use, carrying a non-tagged version of the protein, imposing the use of specific antibodies, which was an advantage since a fluorescent protein tag could interfere with the correct folding/localization. In the first approach, cardosin A was localized by fluorescence microscopy, allowing a general view of its distribution in each tissue. Additionally, electron microscopy techniques were used to improve the resolution of its subcellular localization. High-pressure cryofixation and cryo-substitution were used as they have proven to give better results than conventional chemical fixation. In this work, it has been shown that cardosin A accumulates in the PSV of transgenic Arabidopsis seedlings and is also detected in association with other endomembrane compartments such as the ER, Golgi, or electron-transparent compartments found in the cytoplasm. During this work, only immunofluorescence was obtained for the 24-h germinated seedlings, but we did note the localization in both tissues at this stage. In 24-h germinated seeds, cardosin A localization in the radicle was inconclusive, but the labeling in cotyledons is similar to the anti-PSI labeling surrounding structures that seem to be globoid-like structures/lytic cavities, which could be due to the vigorous remodeling of the vacuolar system that occurs during seed germination, and the PSVs have been suggested to develop into LVs. This could also point to a putative association of the PSI with membranes. Nevertheless, due to this identical localization, it is possible to say that we are detecting the precursor form of the protein, since, in the mature protein, the PSI is cleaved out. In contrast, in the 3-day-old seedling stage, cardosin A was detected in the PSV, but using an anti-PSI antibody, the labeling was detected in vesicles in the cytoplasm. Most probably, the form detected in PSVs is the mature, processed form of the enzyme, indicating that the processing has already occurred in the PSV (Bolte et al., 2011). Nevertheless, there is still labeling with both anti-cardosin A and anti-PSI in small dot-shaped compartments in the cytoplasm, which indicates that more than one form of the protein is present at the same time but in different compartments within the cell. When using the Arabidopsis heterologous system, the localization of cardosin A and cardosin B is, however, different: the light and electron microscopy results show that cardosin A accumulates in PSVs in the early germination steps of transgenic Arabidopsis. In a parallel study on Arabidopsis expressing cardosin B under the same expression system and in the same germination stages, cardosin B was localized in the cell wall, and not detected in the PSVs at any time point (unpublished results). This observation is consistent with the results obtained by da Costa et al. (2010) on protoplasts from *N. tabacum* leaves, where cardosin B was finally secreted as well, leading the authors to propose a role for the cell wall in regulating intracellular sorting. In all cases, Arabidopsis seems to be potentially interesting as a heterologous system to explore the dual targets of cardosin A and cardosin B. This would not only provide interesting results, considering their localization and trafficking, but also give more insights into the comprehension of their physiological roles in the plant.

### 4.2 PSI putative roles in the cell

**Figure 9:**
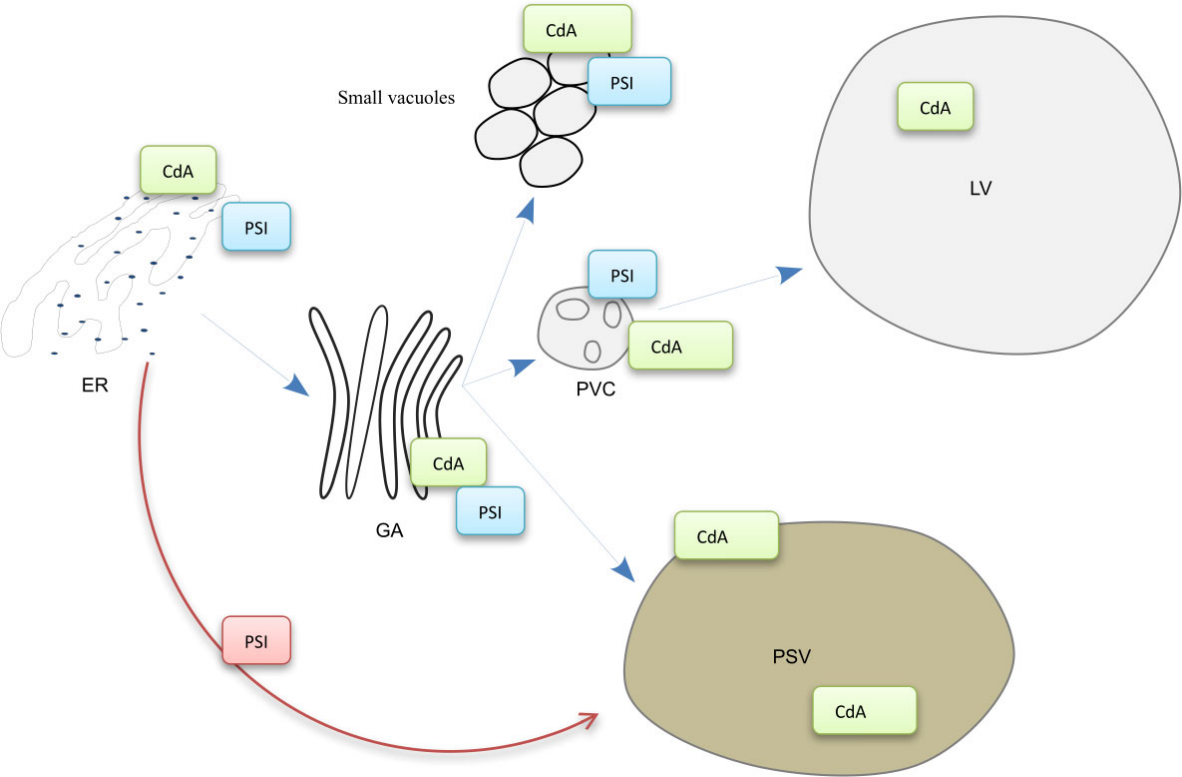
Model for cardosin A biogenesis and putative trafficking pathways in Arabidopsis young seedlings. The labeling of the anti-cardosin A and anti-PSI antibodies used in this study is represented in the scheme. Cardosin A precursor form was detected in the periphery of small vacuoles, and associated with the ER, Golgi, and PVC membranes. The mature form was detected inside PSVs and large central vacuoles. CdA, cardosin A; PSI, plant-specific insert; ER, endoplasmic reticulum; GA, Golgi apparatus; PVC, prevacuolar compartment; LV, lytic vacuole; PSV, protein storage vacuole.

Procardosins are known to be active and may play a part in the proteolysis and processing of storage proteins. Procardosin A’s role in seeds may be related to the hypothesized function of the PSI in membrane lipid conversion during water absorption and solute leakage in active tissues (Pissarra et al., 2007; Muñoz et al., 2014; Zhao et al., 2020). This is consistent with the dual function of aspartic proteinase precursor molecules, which have both a protease and a membrane-destabilizing domain (Muñoz et al., 2014). In contrast with other aspartic proteinases, cardosin A does not have the lysine/tyrosine residues responsible for the inactivation of the precursor enzyme, a fact that could explain the PSI involvement during these events. The results obtained here using the anti-PSI antibody show almost in all cases co-localization with the anti-cardosin A antibody, indicating that it is the precursor form of the enzyme being detected with both antibodies (**Figure 9**) (Bolte et al., 2011). The functions of the PSI, both in the context of the proenzyme and after cleavage, remain elusive, except for a role in the vacuolar targeting of plant AP precursors. Several *in vitro* studies using the PSI domains of different APs have shown that the PSI has the ability to interact with the plasma membranes, inducing their permeabilization and content release (Egas et al., 2002; Muñoz et al., 2010), in a pH-dependent process that also depends on the lipid composition (Egas et al., 2002). Moreover, the PSI crystal structure may oscillate between an open and closed state according to pH (Bryksa et al., 2011). Despite the fact that these roles have not been proven yet in planta, indirect evidence from this and other studies seems to corroborate them, along with the fact that endomembrane compartments vary in membrane composition and pH, which decrease from the ER to the vacuole. The structural changes observed *in vitro* may have a parallel in the PSI-mediated transport from the ER to the vacuole, and its interaction with the membranes can be crucial for the process. In fact, the PSI-mediated transport to the vacuole may be related to its ability to interact with membranes rather than its interaction with a vacuolar receptor, as described for other VSDs. Bryksa and co-workers (Bryksa et al., 2011) proposed a model in which the PSI would change from closed to open after being released from the proenzyme, facilitating its interaction with the bilayer membrane. The drop in pH would promote membrane association and dimerization, bringing the two membranes together and promoting their fusion. The fusogenic activity of the PSI has been largely discussed; however, to date, it has only been shown to happen *in vitro*, with only indirect hints from *in vivo* studies. As a whole, alongside its role in mediating protein transport, the PSI can also have enzymatic activity, independent of the proenzyme, being considered an enzyme inside an enzyme (Bryksa et al., 2011).

### 4.3 Cardosin A is able to bypass BFA blockage via a PSI-dependent route

Several APs were detected and isolated from seeds (for review (Mutlu and Susannah, 1999; Simões and Faro, 2004),) and were well characterized in terms of processing and proteolytic activity, but very little data are available on their intracellular sorting and routes. Until now, phytepsin is the only AP for which this has been attempted. Using pulse-chase, endo-glycosidase H, and inhibitors, Glathe et al. (1998) showed that phytepsin processing events occur along the secretory pathway to the PSV, passing through the Golgi and some post-Golgi compartments, not identified by the authors at the time. In this study, we started to uncover cardosin A trafficking to PSVs in Arabidopsis seedlings using BFA to block the trafficking between the ER and Golgi. We gave a 24-h interval between the addition of the chemical inductor and the BFA because BFA is known to block germination (Geldner and Palme, 2001). In this research, cardosin A was always found in association with the Golgi, even when the BFA caused the disruption of its vesicles, meaning that cardosin A associates with the Golgi in this system. This is consistent with the data presented by Duarte et al. (2008) that showed that cardosin A was partially endo-glycosidase H resistant in Arabidopsis seedlings. The results obtained in this study show that some proteins had already reached the PSV at the beginning of the assay, despite the fact that the majority was still in intermediate compartments of the secretory system. We did quantify cardosin A expression by TEM, by counting cardosin A gold dots inside PSVs in several micrographs. The results showed that the amount of gold dots inside the PSVs increases with time, indicating that part of the protein can escape the BFA blockage. It is known that cardosin A could bypass the GA on its route to the PSV and that this bypass is PSI-dependent. However, to date, there was no clear evidence regarding such a pathway *in vivo* or the context of non-mutated cardosin A, as studies have been conducted in leaves (either in Arabidopsis or in *N. tabacum*), which only have a large LV (Duarte et al., 2008; da Costa et al., 2010; Pereira et al., 2013). Additionally, it has been shown that in *N. tabacum* leaves although cardosin A has a poly-sorting mechanism in the vacuole and the PSI can mediate a Golgi-independent route, the two vacuolar signals are not activated simultaneously, and the C-terminal one is dominant over the PSI, at least in that system. Pereira et al. (2013) discussed the possibility that this poly-sorting mechanism could be an adaptation of the plants and that the PSI-mediated route would be activated in specific situations, namely, in tissues under high metabolic activity as the flowers and seeds, where cardosin A was observed to accumulate in high amounts (Ramalho-Santos et al., 1997; Pereira et al., 2008; Pereira et al., 2013). The results obtained in this work seem to support this hypothesis, as cardosin A was still accumulating in PSVs after ER-to-Golgi transport was blocked by BFA. As cardosin A C-terminal-mediated transport was retained in BFA compartments, one hypothesis is that cardosin A trafficking to the PSV is being mediated by the PSI region. This work also presents interesting documentation of the structural effects on the Golgi and TGN after prolonged exposure to BFA up to 20 h. This reopens other questions still being debated on the Golgi and TGN disassembly and reformation while maintaining motility and protein transport. More importantly, it shows how BFA remains an important tool for uncovering controversial questions at the intersection of the endocytic and vacuolar pathways.

## Data availability statement

The original contributions presented in the study are included in the article/**Supplementary Material**. Further inquiries can be directed to the corresponding author.

## Author contributions

Conceptualization, CP, JP and SP. Methodology, CP. Investigation, CP. Data analysis, CP, VV, JP, and SP. Writing—original draft, CP, and SP. Writing—review and editing, CP, VV, JP, and SP. Funding acquisition, CP, JP, and SP. All authors contributed to the article and approved the submitted version.

## Supplementary material

Supplementary Figure 1 Immunofluorescence of cardosin A and PSI in radicle and cotyledon sections of the Arabidopsis-inducible expression system 24 h after seed germination. **(A)** Stages after seed germination: 24 h, 48 h, and 72 h after germination. **(B)** Immunofluorescence of cardosin A in radicle and cotyledon sections of Arabidopsis inducible expression system using a polyclonal antibody against cardosin 24 h after seed germination. In radicle sections, labeling was detected associated with membranes in the cytoplasm (B - **a, b**, white arrowheads); in cotyledon sections, cardosin A was detected in the cytoplasm (B - **c, d**, white arrowheads) and associated with a globoid-like structure/lytic cavities (yellow arrowheads). **(C)** Immunofluorescence using an anti-PSI antibody in radicle and cotyledon sections of the Arabidopsis-inducible expression system 24 h after seed germination. In radicle sections labeling was detected in the cytoplasm and associated with globoid-like structure/lytic cavities (a, b, white arrowheads); in cotyledon sections, the PSI was also detected in the cytoplasm and associated with the globoids’ membrane (c, d, white arrowheads). Scale bars: a, c, 45 µm; c, d 9 µm.

Supplementary Figure 2 Immunolocalization of γ-TIP in sections of Arabidopsis expressing cardosin A under an inducible expression system. (a–c) Immunogold labeling coupled to a 10 nm gold-conjugated secondary antibody. Labeling of γ -TIP was detected in the periphery of protein storage vacuoles and also in the periphery of some vesicles in the cytoplasm (red circles). Scale bars: a, b, 200 nm.

Supplementary Figure 3 Ultrastructural observations of brefeldin A (BFA) effect on Arabidopsis radicle cells expressing cardosin A. Micrographs of cell ultrastructure obtained from 3-day-old seedlings after cryofixation. Epoxy resin sections of dexamethasone-induced radicles evidencing the general organization of the cell. (a–c) 0 h treatment; (d–f) 2 h treatment; (g–i) 4 h treatment; (j–l) 8 h treatment; (m–o) 20 h treatment. Example of the recognizable polarity of the Golgi stack from *cis* (c) to *trans* cisternae (t) (b). Scale bars: 400 nm.

Supplementary Figure 4 Control (solvent-only) for the BFA treatment using only dimethyl sulfoxide (DMSO) to detect any structural effect of the solvent in the cells. No effect in the Golgi bodies or TGN was observed when compared to the drastic changes observed for BFA-treated samples and cardosin A localization was similar to that of BFA-treated cells (red circles). (a–c) 2 h treatment; (d–f) 4 h treatment; (g–i) 8 h treatment; (j–l) 20 h treatment. Scale bars: 200 nm.

Supplementary Figure 5 Immunogold labeling of PSI in radicle sections of the Arabidopsis-inducible expression system 0, 2, 4, 8, and 20 h upon addition of BFA. **(A)** The exact time of BFA addition to the medium (0 h) was used as a control (a–c). PSI labeling was detected in association with the same compartments as for cardosin A BFA-treated cells, but it seems that there is less detection of PSI (red circles). Note the horseshoe Golgi bodies that, towards the prolonged exposure, completely surround other organelles, which indicates that the Golgi bodies’ cisternal rims might have fused together, and this compartment could also be an ER-Golgi hybrid at this point. PSI was also detected in association with vesicles closely associated with the ER and in proximity to the Golgi bodies, which could be ER-derived carriers. Red circles mark the gold labeling of PSI. Scale bars: a-e, g-o, 200 nm; f, 100 nm.
