# Supplementary figures and images for: The journey of cardosin A in young Arabidopsis seedlings leads to evidence of a Golgi-independent pathway to the protein storage vacuole
Source: Front Plant Sci. 2023 Jul 7;14:1085898. doi: 10.3389/fpls.2023.1085898 (PMC10360190; doi:10.3389/fpls.2023.1085898)

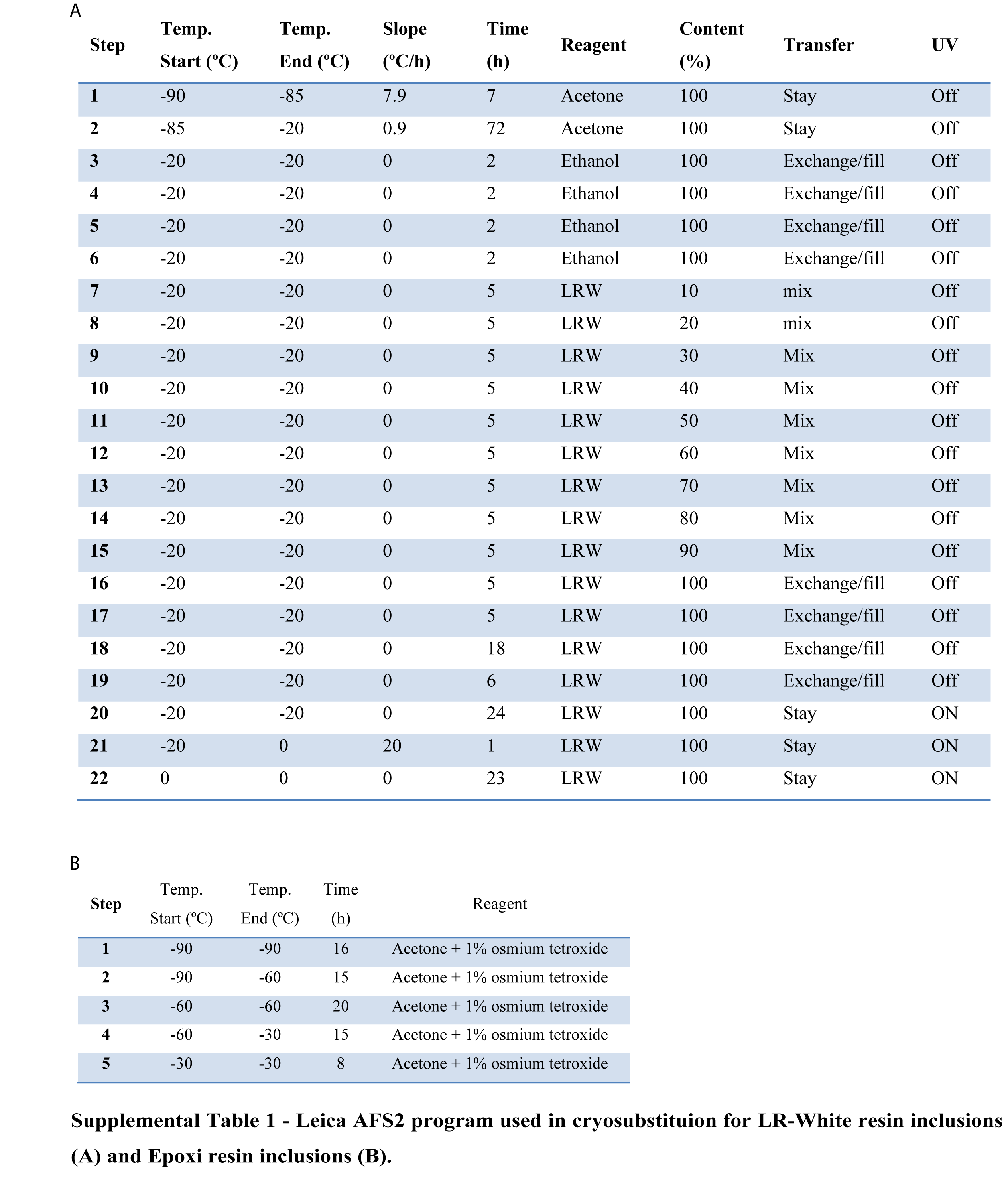

Supplement: Supplementary file 1 [file Image_6.tif]

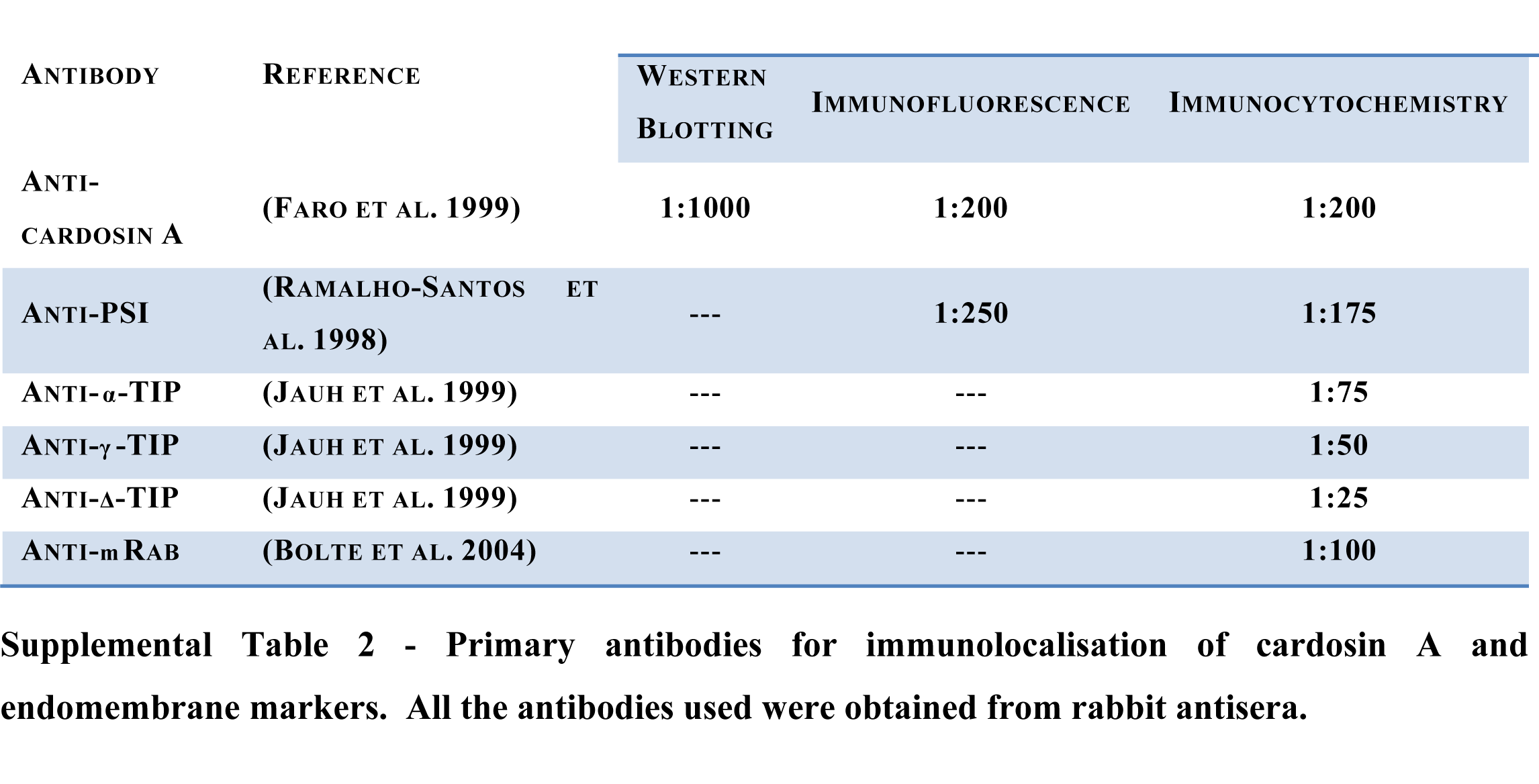

Supplement: Supplementary file 2 [file Image_7.tif]

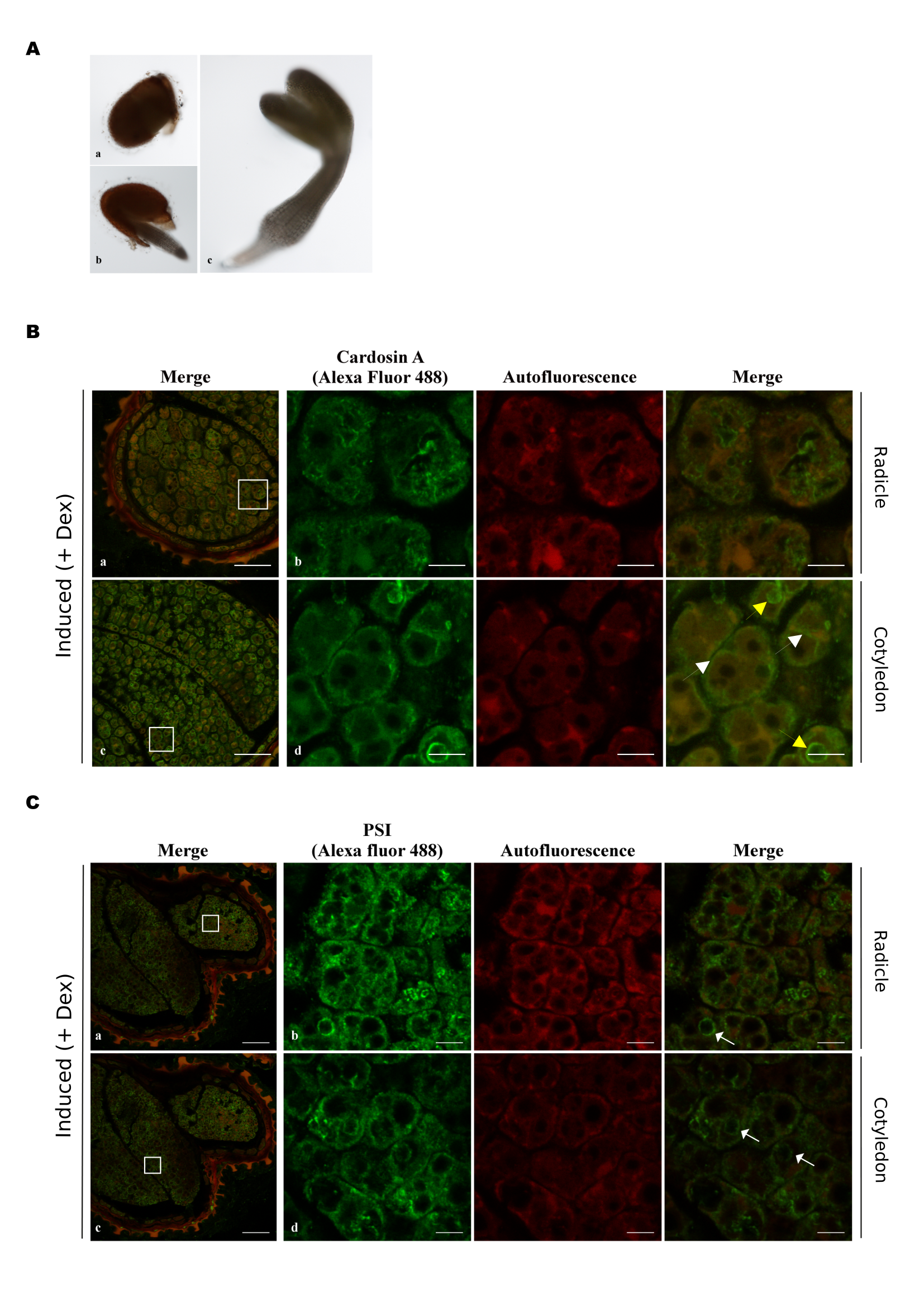

Supplement: Supplementary Figure 1 — Immunofluorescence of cardosin A and PSI in radicle and cotyledon sections of the Arabidopsis-inducible expression system 24 h after seed germination. (A) Stages after seed germination: 24 h, 48 h, and 72 h after germination. (B) Immunofluorescence of cardosin A in radicle and cotyledon sections of Arabidopsis inducible expression system using a polyclonal antibody against cardosin 24 h after seed germination. In radicle sections, labeling was detected associated with membranes in the cytoplasm (B - a, b, white arrowheads); in cotyledon sections, cardosin A was detected in the cytoplasm (B - c, d, white arrowheads) and associated with a globoid-like structure/lytic cavities (yellow arrowheads). (C) Immunofluorescence using an anti-PSI antibody in radicle and cotyledon sections of the Arabidopsis-inducible expression system 24 h after seed germination. In radicle sections labeling was detected in the cytoplasm and associated with globoid-like structure/lytic cavities (a, b, white arrowheads); in cotyledon sections, the PSI was also detected in the cytoplasm and associated with the globoids’ membrane (c, d, white arrowheads). Scale bars: a, c, 45 µm; c, d 9 µm. [file Image_1.tiff]

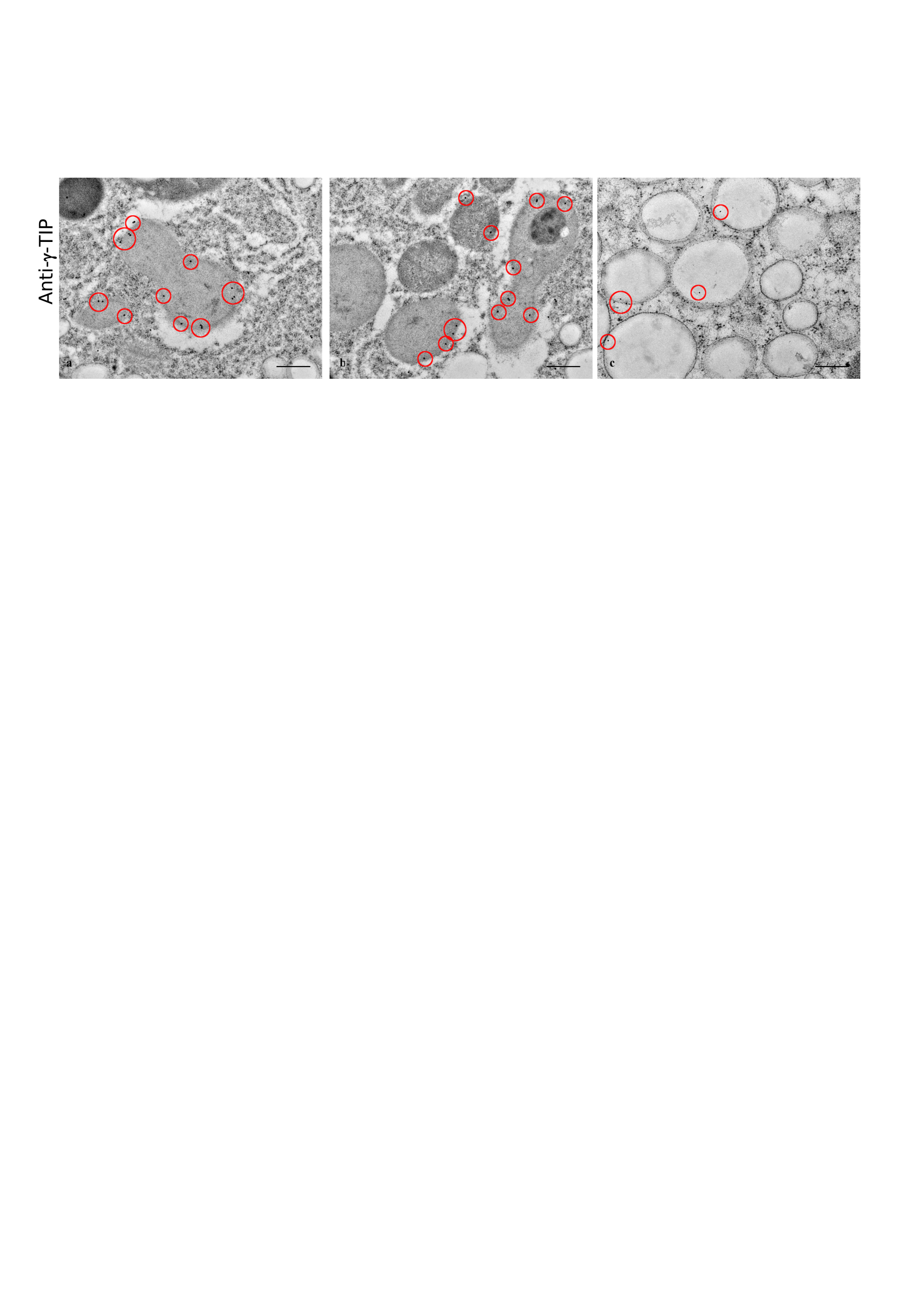

Supplement: Supplementary Figure 2 — Immunolocalization of γ-TIP in sections of Arabidopsis expressing cardosin A under an inducible expression system. (a–c) Immunogold labeling coupled to a 10 nm gold-conjugated secondary antibody. Labeling of γ -TIP was detected in the periphery of protein storage vacuoles and also in the periphery of some vesicles in the cytoplasm (red circles). Scale bars: a, b, 200 nm. [file Image_2.tiff]

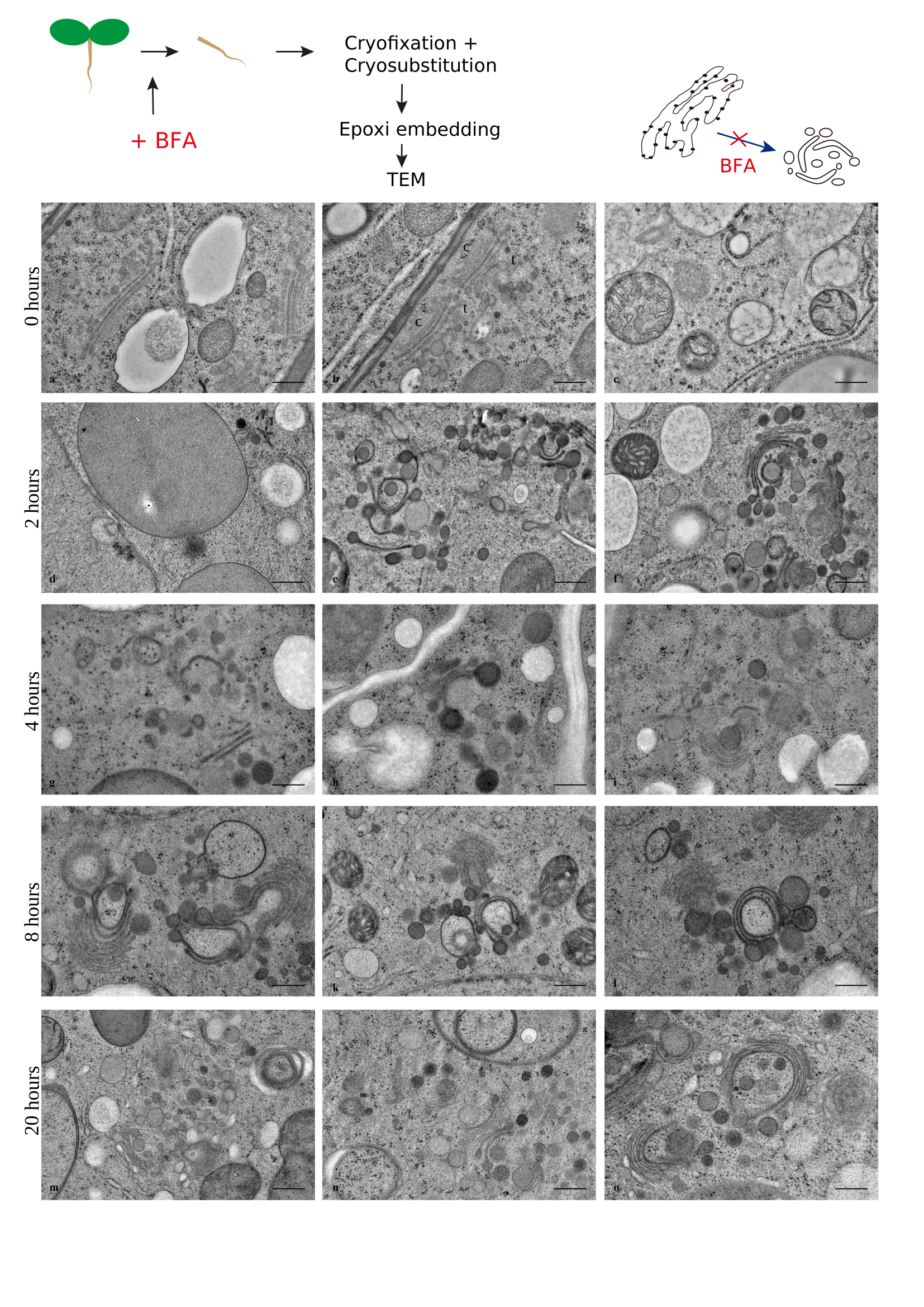

Supplement: Supplementary Figure 3 — Ultrastructural observations of brefeldin A (BFA) effect on Arabidopsis radicle cells expressing cardosin A. Micrographs of cell ultrastructure obtained from 3-day-old seedlings after cryofixation. Epoxy resin sections of dexamethasone-induced radicles evidencing the general organization of the cell. (a–c) 0 h treatment; (d–f) 2 h treatment; (g–i) 4 h treatment; (j–l) 8 h treatment; (m–o) 20 h treatment. Example of the recognizable polarity of the Golgi stack from cis (c) to trans cisternae (t) (b). Scale bars: 400 nm. [file Image_3.tiff]

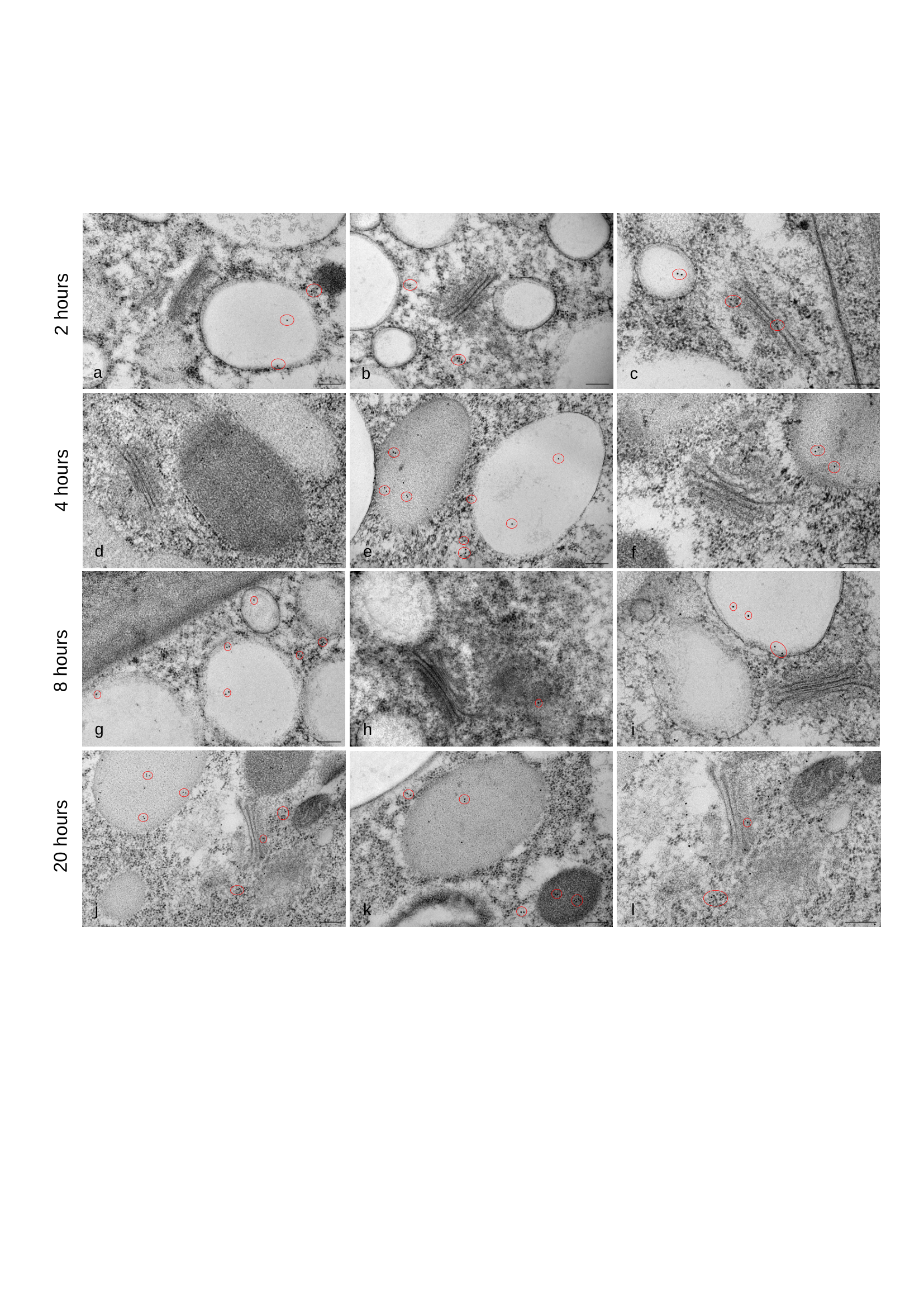

Supplement: Supplementary Figure 4 — Control (solvent-only) for the BFA treatment using only dimethyl sulfoxide (DMSO) to detect any structural effect of the solvent in the cells. No effect in the Golgi bodies or TGN was observed when compared to the drastic changes observed for BFA-treated samples and cardosin A localization was similar to that of BFA-treated cells (red circles). (a–c) 2 h treatment; (d–f) 4 h treatment; (g–i) 8 h treatment; (j–l) 20 h treatment. Scale bars: 200 nm. [file Image_4.tiff]

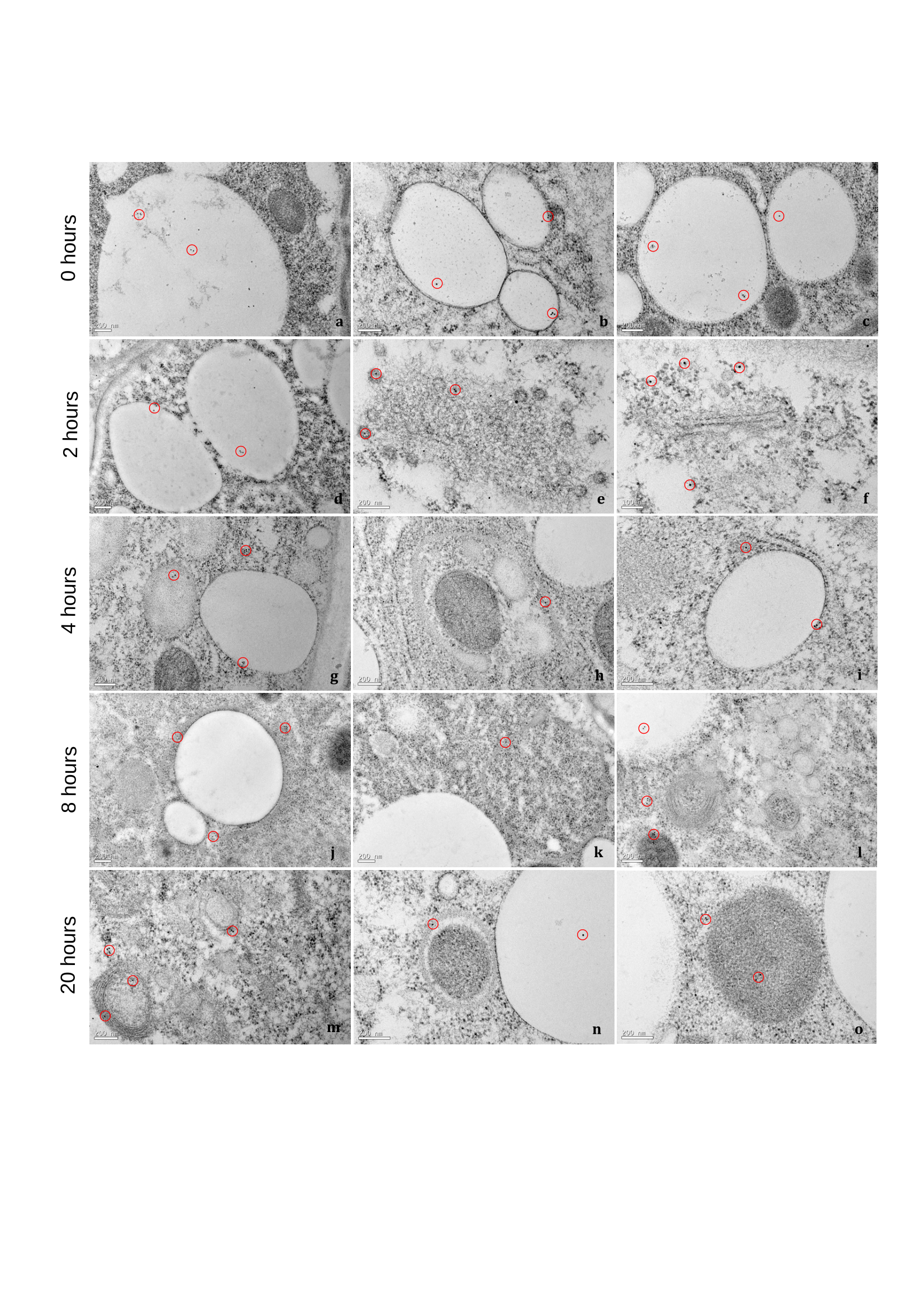

Supplement: Supplementary Figure 5 — Immunogold labeling of PSI in radicle sections of the Arabidopsis-inducible expression system 0, 2, 4, 8, and 20 h upon addition of BFA. (A) The exact time of BFA addition to the medium (0 h) was used as a control (a–c). PSI labeling was detected in association with the same compartments as for cardosin A BFA-treated cells, but it seems that there is less detection of PSI (red circles). Note the horseshoe Golgi bodies that, towards the prolonged exposure, completely surround other organelles, which indicates that the Golgi bodies’ cisternal rims might have fused together, and this compartment could also be an ER-Golgi hybrid at this point. PSI was also detected in association with vesicles closely associated with the ER and in proximity to the Golgi bodies, which could be ER-derived carriers. Red circles mark the gold labeling of PSI. Scale bars: a-e, g-o, 200 nm; f, 100 nm. [file Image_5.tiff]
